# Functional transcriptome analysis of the postnatal brain of the Ts1Cje mouse model for Down syndrome reveals global disruption of interferon-related molecular networks

**DOI:** 10.1186/1471-2164-15-624

**Published:** 2014-07-22

**Authors:** King-Hwa Ling, Chelsee A Hewitt, Kai-Leng Tan, Pike-See Cheah, Sharmili Vidyadaran, Mei-I Lai, Han-Chung Lee, Ken Simpson, Lavinia Hyde, Melanie A Pritchard, Gordon K Smyth, Tim Thomas, Hamish S Scott

**Affiliations:** Genetics and Regenerative Medicine Research Centre, Faculty of Medicine and Health Sciences, Universiti Putra Malaysia, 43400 UPM Serdang, Selangor, Malaysia; Walter and Eliza Hall Institute of Medical Research, 1G Royal Parade, Parkville, Victoria 3052 Australia; Department of Obstetrics and Gynaecology, Faculty of Medicine and Health Sciences, Universiti Putra Malaysia, 43400 UPM Serdang, Selangor, Malaysia; Pathology Department, The Peter MacCallum Cancer Centre, East Melbourne, Victoria, 3002 Australia; Department of Human Anatomy, Faculty of Medicine and Health Sciences, Universiti Putra Malaysia, 43400 UPM Serdang, Selangor, Malaysia; Department of Pathology, Faculty of Medicine and Health Sciences, Universiti Putra Malaysia, 43400 UPM Serdang, Selangor, Malaysia; Department of Biochemistry and Molecular Biology, Monash University, Melbourne, Victoria 3800 Australia; Department of Molecular Pathology, SA Pathology and Centre for Cancer Biology, P.O. Box 14, Rundle Mall Post Office, Adelaide, South Australia 5000 Australia; School of Medicine, Faculty of Health Sciences, University of Adelaide, Adelaide, South Australia 5005 Australia

## Abstract

**Background:**

The Ts1Cje mouse model of Down syndrome (DS) has partial triplication of mouse chromosome 16 (MMU16), which is partially homologous to human chromosome 21. These mice develop various neuropathological features identified in DS individuals. We analysed the effect of partial triplication of the MMU16 segment on global gene expression in the cerebral cortex, cerebellum and hippocampus of Ts1Cje mice at 4 time-points: postnatal day (P)1, P15, P30 and P84.

**Results:**

Gene expression profiling identified a total of 317 differentially expressed genes (DEGs), selected from various spatiotemporal comparisons, between Ts1Cje and disomic mice. A total of 201 DEGs were identified from the cerebellum, 129 from the hippocampus and 40 from the cerebral cortex. Of these, only 18 DEGs were identified as common to all three brain regions and 15 were located in the triplicated segment. We validated 8 selected DEGs from the cerebral cortex (*Brwd1*, *Donson*, *Erdr1*, *Ifnar1*, *Itgb8*, *Itsn1*, *Mrps6* and *Tmem50b*), 18 DEGs from the cerebellum (*Atp5o*, *Brwd1*, *Donson*, *Dopey2*, *Erdr1*, *Hmgn1*, *Ifnar1*, *Ifnar2*, *Ifngr2*, *Itgb8*, *Itsn1*, *Mrps6*, *Paxbp1*, *Son*, *Stat1*, *Tbata, Tmem50b* and *Wrb*) and 11 DEGs from the hippocampus (*Atp5o*, *Brwd1*, *Cbr1*, *Donson*, *Erdr1*, *Itgb8*, *Itsn1*, *Morc3*, *Son*, *Tmem50b* and *Wrb*). Functional clustering analysis of the 317 DEGs identified interferon-related signal transduction as the most significantly dysregulated pathway in Ts1Cje postnatal brain development. RT-qPCR and western blotting analysis showed both Ifnar1 and Stat1 were over-expressed in P84 Ts1Cje cerebral cortex and cerebellum as compared to wild type littermates.

**Conclusions:**

These findings suggest over-expression of interferon receptor may lead to over-stimulation of Jak-Stat signaling pathway which may contribute to the neuropathology in Ts1Cje or DS brain. The role of interferon mediated activation or inhibition of signal transduction including Jak-Stat signaling pathway has been well characterized in various biological processes and disease models including DS but information pertaining to the role of this pathway in the development and function of the Ts1Cje or DS brain remains scarce and warrants further investigation.

**Electronic supplementary material:**

The online version of this article (doi:10.1186/1471-2164-15-624) contains supplementary material, which is available to authorized users.

## Background

Down Syndrome (DS) is a genetic disorder resulting from trisomy or partial trisomy of human chromosome 21 (HSA21). This syndrome is a non-heritable genetic disorder that occurs at a prevalence of approximately 1 in 750 live births [1]. DS has been associated with more than 80 clinical manifestations, including cognitive impairment or intellectual disability, craniofacial features, cardiac abnormalities, hypotonia and early onset Alzheimer’s disease [2, 3]. In terms of cognitive impairment, DS individuals have an average Intelligence Quotient (IQ) value of 50 [4] as well as learning impairment involving both long-term and short-term memory [5]. DS individuals also present with reduced brain size, brain weight, brain volume, neuronal density, and neuronal distribution with neurons that are characterized by shorter dendritic spines, reduced dendritic arborization and synaptic abnormalities [6–8].

There are various hypotheses that attempt to explain the genotype-phenotype relationship of DS. The gene dosage imbalance hypothesis states that an increased copy number of genes on HSA21 leads to an overall increase in gene and protein expression and a subset of these directly result in the traits associated with DS [1]. In contrast, the amplified developmental instability hypothesis suggests that the dosage imbalance of genes on HSA21 results in a general disruption of genomic regulation and expression of genes involved in development, which upsets normal homeostasis and results in many of the traits associated with DS [9]. A further proposed hypothesis is known as the critical region hypothesis and is based on genetic analyses performed on individuals with partial trisomy of HSA21. This line of thinking suggests that a small set of genes within the Down Syndrome Critical or Chromosomal Region (DSCR) are responsible for the development of common DS phenotypes [10]. However, this hypothesis is not supported by experiments on DS individuals, which demonstrated that the DSCR is more likely to be a susceptible region for DS phenotypes, rather than a single critical region causing all DS phenotypes [11–13]. In reality, it is unlikely that the DS traits are caused by one genetic mechanism but instead are due to a combination of mechanisms, with the added complexity of further genetic and epigenetic controls [14]. Some researchers have suggested that dosage imbalance of certain genes may not have any effect on the DS phenotype as they are “dosage compensated” under certain circumstances [1].

Significant genetic homology exists between HSA21 and mouse chromosome 16 (MMU16) [15], MMU17 and MMU10 [16], which has allowed the generation of mouse models of DS and testing of genotype-phenotype correlation hypotheses. There are a few strains of mice that are trisomic for segments of MMU16 that are homologous to HSA21 including Ts65Dn [mitochondrial ribosomal protein L39, (*Mrpl39*)-zinc finger protein 295, (*Znf295*)] [17], Ts1Yey [RNA binding motif protein 11, (*Rbm11*)-*Znf295*] [18], Ts1Cje [superoxide dismutase 1, soluble, (*Sod1*)-*Znf295*] [19] and Ts1Rhr [carbonyl reductase 1, (*Cbr1*)- myxovirus (influenza virus) resistance 2, (*Mx2*)] [12] strains. In addition, the Ts2Yey [protein arginine N-methyltransferase 2, (*Prmt2*)-pyridoxal (pyridoxine, vitamin B6) kinase, (*Pdxk*)] strain [20] is trisomic for MMU10 segments, whereas the Ts3Yey [ribosomal RNA processing 1 homolog B (S. cerevisiae), (*Rrp1b*)-ATP-binding cassette, sub-family G (WHITE), member 1, (*Abcg1*)] [20] and Ts1Yah [U2 small nuclear ribonucleoprotein auxiliary factor (U2AF) 1, (*U2af1*)-*Abcg1*] [21] strains are trisomic for segments of MMU17. Each of these mouse models was found to perform differently in cognitive and hippocampal long-term potentiation (LTP) or long-term depression (LTD) tests and exhibit differences in brain morphology and behavioural phenotypes as well as neuropathology [22]. As such, there is currently no perfect mouse model to study the DS brain. In 2010, Yu and colleagues [20] generated a mouse model [Dp(10)1Yey/+;Dp(16)1Yey/+;Dp(17)1Yey/+] with regions that are syntenic to all of HSA21. This mouse model is characterised by several DS-related neuropathological features including cognitive impairment and reduced hippocampal LTP. Unfortunately, the mice develop hydrocephalus, a phenotype that is rarely associated with DS, and 25% of these animals die between 8 to 10 weeks of age [20].

The Ts1Cje mouse model, also known as T(12;16)1Cje, was developed in 1998 and carries a partial trisomy of MMU16 resulting from a translocation of a segment of MMU16 spanning across the superoxide dismutase 1 (*Sod1*) gene to the zinc finger protein 295 (*Znf295*) gene onto MMU12 [19, 23]. This trisomic region is syntenic to HSA21. Recent literature reports a significant correlation between Ts1Cje mice phenotypes and DS individuals, including altered hippocampus-dependent learning and memory [24–26], craniofacial defects [27] and reduced cerebellar volume [23, 28]. This makes Ts1Cje a suitable model to study the neurobiology networks and mechanisms that contribute to the neuropathology in DS individuals. Olson and colleagues [28] reported that the Ts1Cje mouse is defective in both prenatal and postnatal neurogenesis. We have recently demonstrated that adult Ts1Cje mice start with a similar number of adult neural stem cells as their control littermates, but later develop fewer neuronal progenitors, neuroblasts and neurons [29]. In that study we also reported that differentiated Ts1Cje neurons harbour fewer neurites and have an increased number of astrocytes, which demonstrates that the Ts1Cje mouse has defective neurogenesis and neuronal development. Similar observations have been reported by different studies that showed impaired adult neurogenesis in the subventricular zone (SVZ) and impaired embryonic neurogenesis in Ts1Cje neocortices [30]. The Ts1Cje hippocampus also exhibits abnormal short- and long-term synaptic plasticity [26] as well as an impairment that is restricted to the spatially oriented domain, since short- and long-term novel object recognition memory is conserved [25].

Many genomic studies have been conducted on various tissues from mouse models of DS. To date, gene expression studies on Ts1Cje have mostly been done on the postnatal cerebellum up to day 30 [23, 31, 32]. Gene expression analyses on Ts1Cje whole brain at postnatal day 0 [33], and on neocortical neurospheres at embryonic day 14.5 [34] have also been reported. We have previously analysed the global gene expression in Ts1Cje adult neural stem cells (P84) [29]. All previous studies have been completed on specific brain regions or the whole brain and have not encompassed the entire postnatal brain development period. In addition, gender differences and hormonal influences may also be a confounding factor in some of these gene expression studies as not all reported the gender of their subjects and littermate controls. In order to understand the effect of segmental MMU16 trisomy on the postnatal Ts1Cje brain and the complex mechanisms that may result in neuropathology, we performed a comprehensive spatiotemporal gene expression profiling analysis of 3 brain regions (cerebral cortex, cerebellum and hippocampus) at 4 different time points (Postnatal day (P)1, P15, P30 and P84). These regions were selected for analysis as they are most commonly reported to be affected by neuropathology in DS and mouse models [35]. Furthermore, mice at postnatal day (P)1, P15, P30 and P84, correspond to postnatal brain development and function during the neonatal, juvenile, young adult and adult periods.

## Methods

### Ethics statement, animal breeding, handling and genotyping

Breeding procedures, husbandry and all experiments performed on mice used in this study were carried out according to protocols approved by the Walter and Eliza Hall Institute Animal Ethics Committee (Project numbers 2001.45, 2004.041 and 2007.007) and the Faculty of Medicine and Health Sciences, Universiti Putra Malaysia Animal Care and Use (ACU) committee (Approval reference: UPM/FPSK/PADS/BR-UUH/00416). All sex matched disomic and trisomic littermates involved in the study were generated by mating Ts1Cje males with C57BL/6 female mice. All mice were kept in a controlled environment with an equal light/dark cycle. Unlimited standard pellet diet and water were provided*.* Genomic DNA was extracted from mouse-tails and genotyped using multiplex PCR primers for neomycin (*neo*) and glutamate receptor, ionotropic, kainite 1 (*Grik1*) as an internal control as described previously [19] with substitution of gel electrophoresis with high resolution melting analysis.

### Tissue procurement, RNA extraction, quality control and microarray analysis

Procurement of the cerebral cortex, hippocampus and cerebellum were performed on 3 Ts1Cje and 3 disomic female littermates at 4 time points (P1.5, P15, P30 and P84) according to a method described previously [36]. Only female mice were utilized in the study to avoid the downstream effects of Y-linked genes on neural sexual differentiation [37]. Total RNA was purified from each tissue, with assessment of RNA quality and quantification of purified RNA performed according to methods described previously [29]. Each RNA sample was processed using the Two-Cycle Target Labeling Assay and hybridized onto Affymetrix Gene-Chip® Mouse Genome 430 2.0 arrays (Affymetrix, USA) according to the manufacturer’s protocols. Fluorescent signals were detected using a GeneChip® Scanner 3000 (Affymetrix, USA) and expression data were pre-processed and normalized using the gcRMA algorithm [38]. All datasets were normalized by comparing Ts1Cje trisomic mouse brains to their disomic littermates.

### Differentially expressed genes (DEGs), gene ontology and pathway analyses

The Empirical Bayes t-statistic [39] was used to analyse differential expression of genes between groups according to a method described previously [29]. Briefly, stringent criteria were employed to select differentially expressed genes (DEGs) from the analysis including *t-*statistic values of ≥ 4 or ≤ −4 and an adjusted *P*-value of ≤ 0.05. Selected DEGs were collectively analysed for functional ontologies using the Database for Annotation, Visualisation and Integrated Discovery (DAVID) [40]. High classification stringency was used to analyse the gene lists with the following settings; a kappa similarity threshold of 0.85, a minimum term overlap of three, two initial and final group membership with 0.50 multiple linkage threshold and a modified Fisher-exact *p-*value or enrichment thresholds of 0.05. All DEGs were analysed according to brain regions and/or time-points.

### Quantitative real time polymerase chain reaction (RT-qPCR)

RT-qPCR was performed to validate the expression of DEGs using cDNAs that were generated from the same RNAs used for microarray analysis. First strand cDNA was synthesized from 3000 ng total RNA using random hexamers and the SuperScript™III Reverse Transcriptase Kit (Invitrogen, USA) according to the manufacturer’s protocol. Primers were designed and probes selected using ProbeFinder version 2.34 (except for Stat1 where ProbeFinder version 2.45 was used) at the Universal ProbeLibrary Assay Design Center (Roche Applied Science http://lifescience.roche.com/). RT-qPCR was performed in triplicate using the LC480 Master Probe Mix (Roche Diagnostics, Switzerland) and Universal ProbeLibrary (UPL) probe (Roche Diagnostics, Australia) according to published methods [29, 36] (see Additional file 1 for a full list of primers and UPL probes used). Conditions for the RT-qPCR, calculation of quantification cycle for each signal, determination of PCR efficiencies, reproducibility (R^2^ values) and relative quantification of target gene expression in Ts1Cje and disomic samples were performed essentially according to methods described previously [36]. Successful assays were defined by a PCR efficiency of between 90-110% and an R^2^ values > 0.98.

### Western blotting

Cerebral cortices and cerebella were harvested from 3 adult (P84) Ts1Cje and 3 wild type mice. The samples were homogenised and lysates extracted in 1X radioimmunoprecipitation assay (RIPA) lysis buffer (Millipore, USA) containing protease inhibitor cocktail set III (Calbiochem, USA). Protein concentration was analysed using Coomassie Plus (Bradford) Assay reagent according to manufacturer’s protocol (Thermo Scientific, USA). Protein samples were then separated by 8% SDS-PAGE and Western blots were performed. For immunodetection, the following antibodies were used: anti-Stat1 (#9172; Cell Signaling Technology, USA; 1:200 dilution), anti-Ifnar1 (#127322; Biolegend, USA; 1:200 dilution), anti-Ifnar2 (sc-20218; Santa Cruz, USA; 1:200 dilution), and anti-β-actin (ab8227; Abcam, UK; 1:1000 dilution). Blots were incubated overnight at 4°C with primary antibodies followed by 1 hour incubation at room temperature with HRP-conjugated secondary antibodies. The following secondary antibodies were used: anti-goat (CGHL-50AX809015, ICL. Inc., USA), anti-mouse (sc-2005, Santa Cruz, USA) and anti-rabbit (#406401, Biolegend, USA) (all at 1:2500 dilution). Immunoreactivity was visualized using the WesternBright™ Quantum™ (Advansta Corp., USA) for β-actin and WesternBright™ Sirius™ (Advansta Corp., USA) for Stat1, Ifnar1 and Ifnar2. Pixelation analyses of bands were performed using ImageJ software according to the standard protocol published at http://rsb.info.nih.gov/ij.

## Results

### Microarray datasets and differentially expressed genes (DEGs)

To investigate the effect of partial trisomy on postnatal brain development and function in Ts1Cje mice, we performed 72 whole-genome expression analyses using GeneChip® Mouse Genome 430 2.0 Arrays (Affymetrix, Santa Clara, USA). The analyses encompassed comparison of three brain regions (cerebral cortex, cerebellum and hippocampus) at 4 different time points (Postnatal (P)1, P15, P30 and P84) in Ts1Cje and disomic female mice. These datasets are publicly accessible from the Gene Expression Omnibus website under the series accession number GSE49050 (http://www.ncbi.nlm.nih.gov/geo/query/acc.cgi?acc=GSE49050).

**Figure 1:**
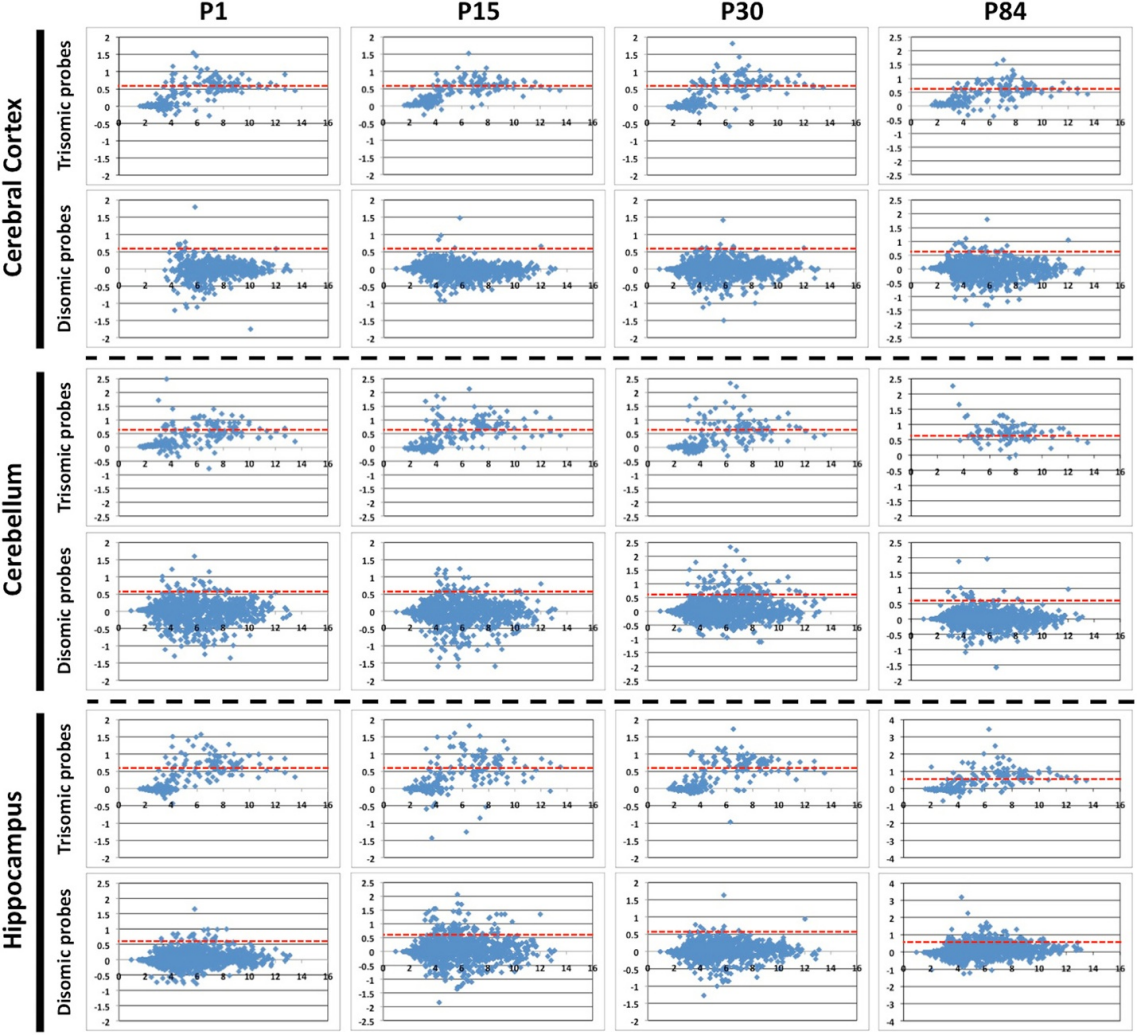
**MA plots of trisomic and disomic microarray probe-sets from 3 different brain regions (cerebral cortex, cerebellum and hippocampus) at 4 postnatal (P) time points (P1, P15, P30 and P84).** The Y-axis represents the M value, which is the ratio (log_2_(T/D)) whereas the X-axis represents the A value, which is the mean ratio (1/2xlog_2_(TxD)). T and D represent the intensities of microarray probe-sets for Ts1Cje and disomic samples, respectively. Each blue dot represents a single probe. Red dotted lines denote the cutoff at M values of 0.58, signifying 1.5-fold upregulation of microarray probe-sets.

To investigate the overall characteristics of genes in the trisomic region, we plotted their log_2_ fold-change (*M*) for trisomic versus disomic mice versus the average log_2_ expression (*A*) (Figure 1). Probe-sets that were not expressed or showed no differences between the groups of mice were plotted near to 0. There was consistently a larger number of probe-sets located in the trisomic region with *M* values greater than 0.58, signifying their 1.5-fold upregulation in various brain regions and developmental stages compared to probe-sets located in disomic regions of the genome. Our observation therefore supports the gene dosage imbalance hypothesis, which specifies that an increased copy number of genes will lead to an overall increase in their expression by 50%.

Genes located within the trisomic region have an increased copy number of 0.5 compared to genes located within disomic regions. According to the gene dosage imbalance hypothesis, we expect only a small fold-change difference in the level of gene expression between Ts1Cje and disomic groups resulting in a small number of globally differentially expressed genes (DEGs) based on our stringent selection criteria (see Methods). The analysis revealed 317 DEGs based on all spatiotemporal comparisons completed between the Ts1Cje and disomic mice (Table 1; Additional file 2). Of these DEGs, 41 are located on the MMU16 triplicated segment (Table 2) and all of the significant probe sets were found to be upregulated by 1.4- – 4.8-fold, which again supports the gene dosage imbalance hypothesis.

**Table 1:** **Summary of microarray analysis**

Time-point Region		P1	P15	P30	P84	Total number of unique DEGs
**Cerebral Cortex**	Probe set	20	5	15	20	40
	DEG	12↑ 8↓	4↑ 1↓	13↑ 2↓	13↑ 6↓	
**Cerebellum**	Probe set	117	53	18	93	201
	DEG	46↑ 66↓	43↑ 1↓	12↑ 4↓	64↑ 23↓	
**Hippocampus**	Probe set	28	59	22	81	129
	DEG	22↑ 4↓	48↑ 3↓	20↑ 1↓	69↑ 7↓	
**Total number of unique DEGs**		131	80	30	145	(317)

↑ denotes ‘upregulation’, ↓ denotes ‘downregulation’, DEG denotes ‘differentially expressed gene’ and P denotes ‘postnatal day’. The value in parentheses denotes non-redundant unique DEGs based on the spatiotemporal comparison between Ts1Cje and disomic mice.

**Table 2:** **Summary of spatiotemporal microarray profiling of 41 DEGs found in the triplicated segment of MMU16**

Full gene name (Official gene symbol)	Probe set ID	Log _2_ expression of Ts1Cje normalized against disomic littermates											
		Cerebral cortex				Cerebellum				Hippocampus			
		P1	P15	P30	P84	P1	P15	P30	P84	P1	P15	P30	P84
RIKEN cDNA 1110004E09Rik gene (1110004E09Rik)	1424315_at	0.72	0.70	0.67	0.73	1.39*	0.91	0.91	0.70	0.61	1.12	0.97	0.83
RIKEN cDNA 2410124H12Rik gene (2410124H12Rik)	1432515_at	0.03	0.09	0.01	0.16	0.07	1.68**	1.51***	2.26***	-0.02	-0.08	0.05	-0.14
ATP synthase, H+ transporting, mitochondrial F1 complex, O subunit (*Atp5o*)	1416278_a_at	0.65	0.49	0.70	0.48	0.43	0.67	0.82*	0.54	0.68	0.37	0.48	1.09***
	1437164_x_at	0.58	0.71	0.72*	0.64	0.68*	0.74**	0.78**	0.59	0.71*	0.64*	0.74*	0.94***
UDP-Gal:betaGlcNAc beta 1,3-galactosyltransferase, polypeptide 5 (*B3galt5*)	1450528_at	0.00	0.02	-0.02	0.00	0.02	0.15	-0.01	0.43	-0.02	-0.11	0.15	1.25*
Expressed sequence BF642829 (BF642829)	1435484_at	0.56	0.34	0.68	-0.04	0.91**	0.75*	0.68	0.01	0.59	0.69	0.89**	0.17
Bromodomain and WD repeat domain containing 1 (*Brwd1*)	1427322_at	0.67	0.45	0.50	0.40	0.32	0.81*	0.84*	0.54	0.78	0.26	0.52	1.11***
	1433955_at	0.55	0.53	0.63	0.86*	1.17***	0.81**	0.57	1.01***	0.39	0.70*	0.70*	0.91***
	1452322_a_at	0.07	0.62	0.47	0.83*	0.14	0.37	0.22	0.94***	0.67*	0.46	0.68*	0.22
C2 calcium-dependent domain containing 2 (*C2cd2*)	1436344_at	0.47	0.47	0.38	0.54	0.27	1.87***	0.47	0.74	0.39	0.71	0.91	0.68
	1437731_at	0.79	0.30	0.15	0.47	0.59	1.21*	0.18	0.45	0.58	0.32	0.55	0.18
Carbonyl reductase 1 (*Cbr1*)	1460196_at	0.67	0.48	0.66	0.58	0.71	0.52	0.30	0.72*	0.64	0.95**	0.72	0.74*
Carbonyl reductase 3 (*Cbr3*)	1427912_at	1.55	1.11	0.82	0.81	1.08	-0.02	0.21	0.79	1.24	2.04*	1.07	0.44
Chromatin assembly factor 1, subunit B (p60) (*Chaf1b*)	1423877_at	1.15***	0.10	0.00	0.08	0.93***	-0.03	-0.06	0.00	1.51***	0.05	-0.08	-0.11
Crystallin, zeta (quinone reductase)-like 1 (*Cryzl1*)	1430547_s_at	0.70	0.57	0.76	0.55	0.60	0.70	0.97	0.94*	0.57	0.17	0.37	1.28***
	1451473_a_at	0.47	0.61	0.86**	0.67	0.61	0.88***	0.49	0.51	0.57	0.82**	0.85**	0.59*
DnaJ (Hsp40) homolog, subfamily C, member 28 (*Dnajc28*)	1420542_at	0.36	0.29	0.14	0.18	0.26	0.33	0.21	0.81*	0.28	0.15	0.26	0.23
Downstream neighbor of SON (*Donson*)	1426739_at	0.76	0.72	0.91**	0.78	0.82*	0.91**	0.75	0.75*	0.81*	0.65	0.63	0.92**
Dopey family member 2 (*Dopey2*)	1428330_at	0.68*	0.47	0.61	0.45	0.58	0.83**	0.70*	0.59	1.00***	0.68*	0.85**	0.77**
Down syndrome cell adhesion molecule (*Dscam*)	1441082_at	0.35	0.70	1.13	0.78	0.41	0.36	0.94	0.74	0.40	1.60**	1.13	0.59
	1449411_at	0.94	0.83	0.98	1.03	0.69	0.54	0.70	1.22*	0.81	1.52**	0.87	0.86
	1458625_at	0.52	0.44	0.74	0.72	0.79	0.11	0.33	0.50	0.72	1.65***	1.39**	0.45
Down syndrome critical region 3 (*Dscr3*)	1415745_a_at	0.87	0.74	0.66	0.91	0.97*	0.93*	0.87	0.66	0.80	0.89*	1.01*	0.94*
E26 avian leukemia oncogene 2, 3' domain (*Ets2*)	1416268_at	0.78	0.62	0.81	0.56	0.54	0.87*	0.65	0.80*	0.62	0.45	0.68	0.77
Phosphoribosylglycinamide formyltransferase (*Gart*)	1416283_at	0.52	0.38	0.92	0.37	0.39	1.17**	0.54	0.93	0.70	0.95	1.20*	0.95
	1424436_at	0.41	0.85	0.70	0.98	0.98	1.03*	0.20	1.30***	0.18	0.93	0.69	0.67
High mobility group nucleosomal binding domain 1 (*Hmgn1*)	1422495_a_at	0.51	0.60	0.59	0.55	0.22	0.96**	0.49	0.68*	0.36	0.16	0.64	0.82**
	1438940_x_at	0.39	0.52	0.50	0.67	0.40	0.76*	0.24	0.54	0.39	0.33	0.50	0.36
	1455897_x_at	0.44	0.69	0.70	0.95	0.49	1.07**	0.30	0.80	0.40	0.52	0.87	0.58
Hormonally upregulated Neu-associated kinase (*Hunk*)	1418260_at	0.66	0.66	0.25	0.68	0.41	0.83	0.87	0.64	1.10*	0.52	0.61	1.16**
Interferon (alpha and beta) receptor 1 (*Ifnar1*)	1442222_at	0.63	0.74	1.05	0.77	0.45	1.12*	0.73	0.68	0.62	0.80	0.80	0.75
	1449026_at	0.80	0.80	1.17*	1.29*	0.55	0.67	0.85	1.10*	0.46	1.12*	0.84	0.75
Interferon (alpha and beta) receptor 2 (*Ifnar2*)	1427691_a_at	0.66	0.52	0.67	0.40	0.55	0.80	0.48	0.58	0.73	1.49*	0.91	0.50
	1440169_x_at	0.72	0.92	0.78	0.60	0.89	0.68	0.45	0.34	1.22	1.49*	0.84	0.62
	1451462_a_at	0.70	0.90	0.99*	0.98	0.61	0.90*	0.80	0.93*	0.81	0.83	0.92*	1.43***
Interferon gamma receptor 2 (*Ifngr2*)	1423558_at	0.31	0.39	0.40	0.44	0.67**	0.14	0.17	0.21	0.21	0.45	0.28	0.26
Intersectin 1 (SH3 domain protein 1A) (*Itsn1*)	1421192_a_at	0.82	0.45	0.90	0.40	0.03	0.94*	1.16**	0.66	1.01*	0.48	0.97*	1.04**
	1425899_a_at	0.58	0.61	0.71	0.22	0.35	0.72	1.37	0.90	0.76	-0.54	0.18	1.67*
	1435885_s_at	0.56	0.61	0.63	0.66**	0.46	1.18*	0.25	0.50	0.49	0.37	0.97	0.21
	1452338_s_at	0.58	0.96	1.42**	1.66	0.44	0.99	0.80	1.30**	0.64	0.25	0.70	0.74
Potassium inwardly-rectifying channel, subfamily J, member 6 (*Kcnj6*)	1425707_a_at	-0.13	0.24	0.22	0.59	0.18	0.29	0.04	0.31	-0.21	0.57	1.07*	-0.06
Microrchidia 3 (*Morc3*)	1420091_s_at	0.55	0.81**	0.64	0.66	0.78**	0.84***	0.69*	0.55	0.64*	0.66*	0.78**	0.99***
	1452224_at	0.69	0.64	0.68	0.66	0.82	0.99	1.21*	0.52	1.20*	0.51	0.55	1.45**
Mitochondrial ribosomal protein S6 (*Mrps6*)	1424440_at	0.84**	0.62	0.73	0.35	0.89**	0.70*	0.89**	1.00***	0.77*	0.72*	0.64	0.91**
	1447585_s_at	0.93	0.74	0.88	0.74	1.13*	0.88	0.53	0.94	0.61	1.15*	0.91	0.61
PAX3 and PAX7 binding protein 1 (*Paxbp1*)	1418007_at	0.55	0.66	0.75	0.59	0.55	1.32*	0.59	0.80	0.83	0.15	0.80	0.56
	1418008_at	0.46	0.38	0.74	0.62	0.56	1.45*	0.63	0.75	0.92	0.21	0.92	0.86
Phosphatidylinositol glycan anchor biosynthesis, class P (*Pigp*)	1436038_a_at	0.69*	0.70	0.77*	0.95**	0.77**	0.75**	0.84**	0.82***	0.38	0.54	0.55	1.16***
PR domain containing 15 (*Prdm15*)	1455459_at	0.41	-0.04	0.47	0.59	0.21	0.37	0.59	0.18	0.44	0.48	0.35	0.95*
Proteasome (prosome, macropain) subunit, alpha type 2 (*Psmg1*)	1448307_at	0.81*	0.52	0.78	0.97*	0.73	0.99**	0.57	0.88**	0.55	1.04**	0.82*	0.94**
Regulator of calcineurin 1 *(Rcan1*)	1416600_a_at	0.98*	0.74	0.89	0.43	0.89	0.71	0.70	0.76	0.73	1.10**	0.84	0.68
Receptor-interacting serine-threonine kinase 4 (*Ripk4*)	1418488_s_at	0.06	0.07	0.00	0.12	0.12	-0.02	0.06	0.02	0.53***	0.02	-0.05	-0.09
Solute carrier family 5 (inositol transporters), member 3 *(Slc5a3)*	1435484_at	0.56	0.34	0.68	-0.04	0.91***	0.75***	0.68	0.01	0.59	0.69	0.89***	0.17
Small integral membrane protein 11(*Smim11*)	1417402_at	0.85	0.43	0.64	0.40	0.90*	0.69	0.59	0.59	0.77	0.37	0.68	0.70
Son cell proliferation protein (*Son*)	1420952_at	0.68	0.69	0.90	0.86	0.36	1.28*	0.71	0.87	0.88	0.54	0.77	0.65
	1435862_at	0.64	0.75	0.80	0.63	0.35	1.04**	1.24***	0.78	0.99*	0.20	0.58	1.17**
	1437924_at	0.63	0.62	0.79	0.82	0.28	0.96**	0.75	0.67	0.35	0.60	0.65	0.73
Synaptojanin 1 (*Synj1*)	1436333_a_at	0.67	0.65	0.77	0.84	0.89	0.57	0.74	0.84	0.74	1.38*	0.79	0.72
	1454961_at	0.60	0.57	0.65	0.63	0.57	0.43	0.58	0.71*	0.56	0.86**	0.55	0.67
Transmembrane protein 50B (*Tmem50b*)	1423707_at	0.78**	0.51	0.70*	0.46	0.83**	0.69*	1.07***	0.71**	0.96***	0.76**	0.84**	0.99***
Tetratricopeptide repeat domain 3 (*Ttc3*)	1416484_at	0.52	0.53	0.68	0.56	0.60	0.39	0.74	0.87*	0.53	0.31	0.26	0.78
	1448361_at	0.45	0.45	0.54*	0.43	0.21	0.43	0.46	0.40	0.33	0.63**	0.45	0.46
URB1 ribosome biogenesis 1 homolog (S. cerevisiae) (*Urb1*)	1454841_at	0.58	0.49	0.77	0.46	0.24	0.16	0.48	0.37	0.98*	0.62	0.76	0.85
Tryptophan rich basic protein (*Wrb*)	1460446_at	0.62	0.64	0.63	0.74	0.72*	0.90*	0.45	0.67*	0.56	0.85**	0.69	0.70*

*p<0.05, **p<0.01 and ***p<0.001 based on Empirical Bayes t-statistic test.

**Figure 2:**
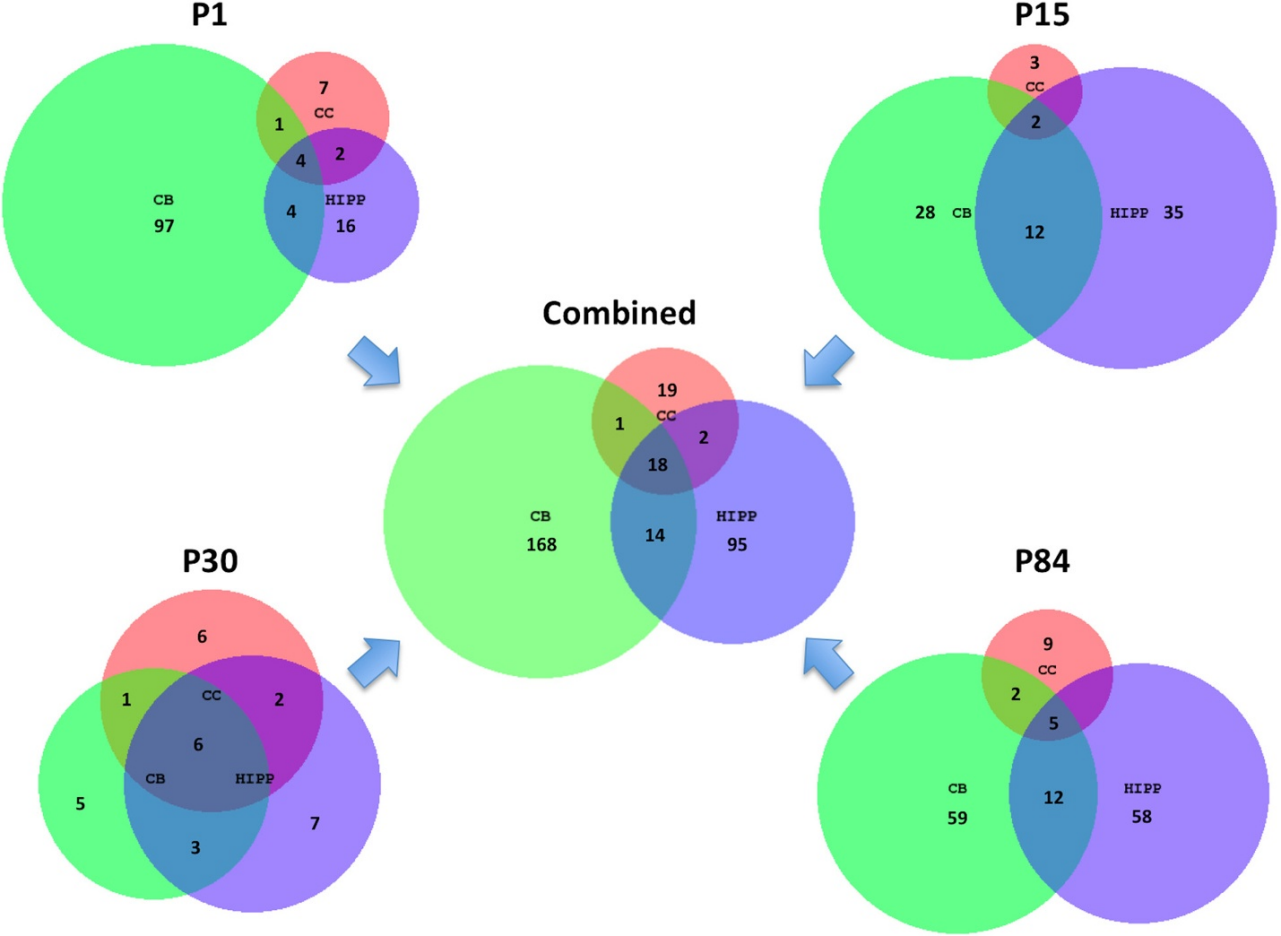
**Venn diagrams depicting the spatiotemporal distribution of DEGs for comparison of Ts1Cje vs.** disomic mice at 4 postnatal (P) time points (P1, P15, P30 and P84). The combined Venn diagram consists of non-redundant DEGs from each brain region at all time points. CC = Cerebral cortex; CB = Cerebellum; HIPP = Hippocampus.

When we considered only spatial comparisons (regardless of time point), 40 DEGs were identified from the cerebral cortex, 201 from the cerebellum and 129 from the hippocampus. Of these DEGs, 16, 33 and 33 were located on the MMU16 triplicated region in the cerebral cortex, cerebellum and hippocampus regions, respectively. We identified 19, 168 and 95 region-specific DEGs for the cerebral cortex, cerebellum and hippocampus, respectively (Figure 2). These observations suggest that the partial trisomy of MMU16 in Ts1Cje mice has a greater effect on gene regulation in the hippocampus and cerebellum as compared to the cerebral cortex. Of all of the DEGs identified, only 18 were found to be common to all three-brain regions [ATP synthase, H + transporting, mitochondrial F1 complex, O subunit, *Atp5o*; bromodomain and WD repeat domain containing 1, *Brwd1*; chromatin assembly factor 1, subunit B (p60), *Chaf1b*; crystallin, zeta (quinone reductase)-like 1,*Cryzl1*; dynein, axonemal, heavy chain 11, *Dnah11*; downstream neighbor of SON, *Donson*; dopey family member 2, *Dopey2*; erythroid differentiation regulator 1, *Erdr1*; interferon (alpha and beta) receptor 1, *Ifnar1*; interferon (alpha and beta) receptor 2, *Ifnar2*; integrin beta 8, *Itgb8*; intersectin 1 (SH3 domain protein 1A), *Itsn1*; microrchidia 3, *Morc3*; mitochondrial ribosomal protein S6, *Mrps6*; phosphatidylinositol glycan anchor biosynthesis, class P, *Pigp*; proteasome (prosome, macropain) assembly chaperone 1, *Psmg1*; transmembrane protein 50B, *Tmem50b* and tetratricopeptide repeat domain 3, *Ttc3*]. Interestingly, 15 out of these 18 DEGs were located in the MMU16 triplicated region (Additional file 2), suggesting that these trisomic genes could be responsible for the global dysregulation of other DEGs within the Ts1Cje brain throughout development.

### Functional clustering of DEGs based on gene ontologies

**Figure 3:**
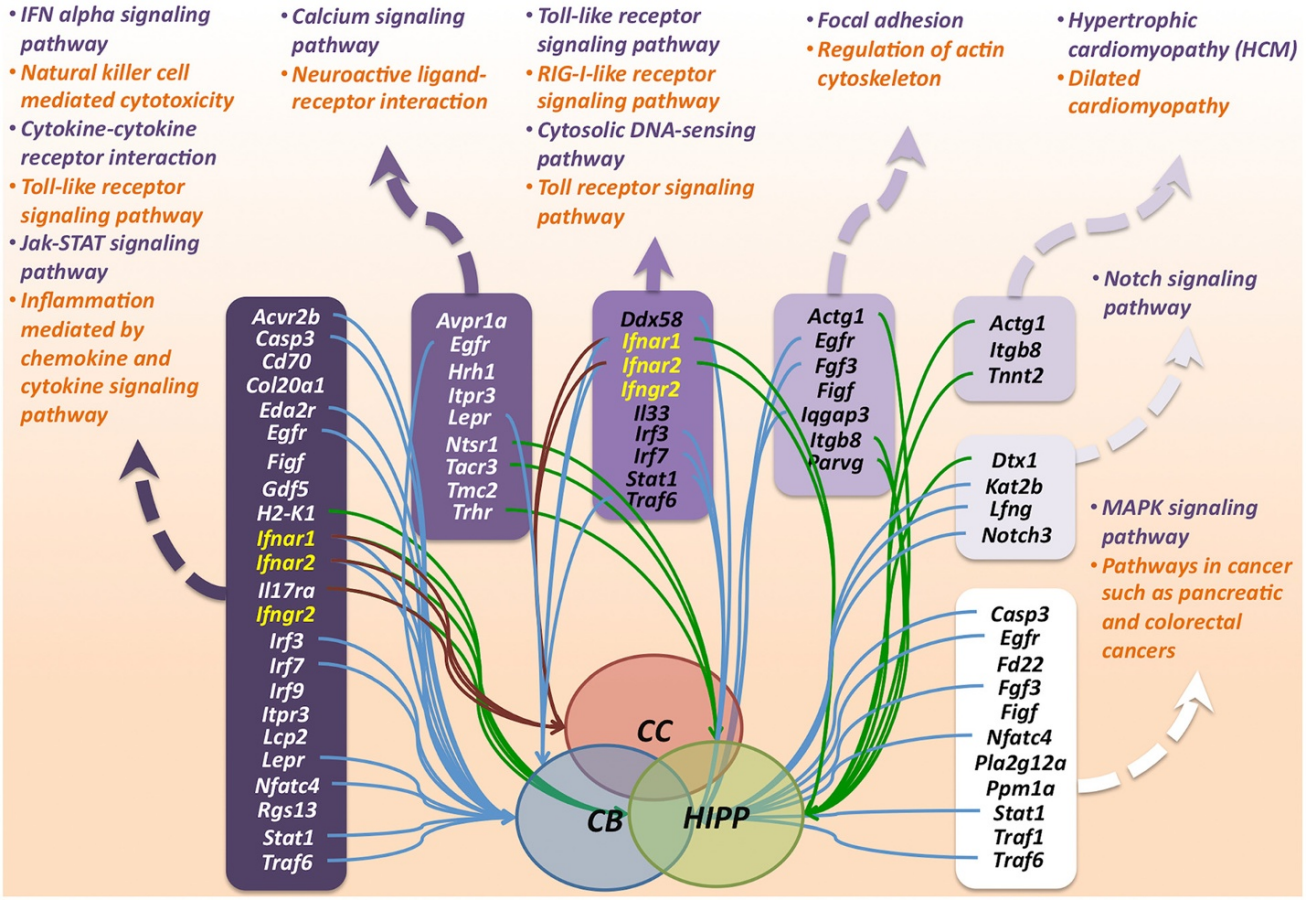
**Summary of functional clustering analysis of 317 DEGs using DAVID tools.** Gene names in yellow denote trisomic genes. Thick dotted lines connect the DEG cluster with their associated functional ontologies whereas the thin solid lines connect DEGs to various brain regions. The colour of the thin solid lines corresponds to the brain regions to which they are connected. CC = Cerebral cortex; CB = Cerebellum; HIPP = Hippocampus.

To dissect the ontologies that are enriched in the list of DEGs, we employed a top-down screening approach to analyze any disrupted molecular networks on a global level, followed by refined analyses involving specific brain regions or developmental stages. An initial analysis of the 317 DEGs revealed 7 significant functional clusters that were associated with interferon-related signaling pathways (23 DEGs, 6 ontologies), innate immune pathways (9 DEGs, 4 ontologies), Notch signaling pathway (4 DEGs, 1 ontology), neuronal signaling pathways (9 DEGs, 2 ontologies), cancer-related pathways (11 DEGs, 4 ontologies), cardiomyopathy-related pathways (3 DEGs, 2 ontologies) and dynamic regulation of cytoskeleton pathways (7 DEGs, 2 ontologies). The functional clustering analysis was repeated using the lists of DEGs from each brain region regardless of developmental stage and subsequently at each developmental stage. The DEGs found at each developmental stage were found to be significantly enriched for the same pathways identified in the list of 317 DEGs (see Additional file 3). The results of the top-down functional screening approach are illustrated in Figure 3.

Based on the analysis involving all 317 DEGs, only 3, namely *Ifnar1*, *Ifnar2* and interferon gamma receptor 2 (*Ifngr2*), from the triplicated MMU16 region were enriched in the functional clusters that were identified (Figure 3). These DEGs were found within two annotation clusters for six interferon-related signaling pathways, including the interferon alpha signaling pathway, natural killer cell mediated cytotoxicity, cytokine-cytokine receptor interaction, toll-like receptor signaling pathway, the Janus kinase (Jak)-signal transducer and activation of transcription (Stat) signaling pathway and the inflammation mediated by chemokine and cytokine signaling pathways. Interestingly, these DEGs are surface interferon receptors and were also found to be enriched for the same functional clusters in all regions of the brain assessed regardless of developmental stage. This suggests that trisomy of *Ifnar1*, *Ifnar2* and *Ifngr2* is crucial in causing dysregulation of interferon-related pathways, which may in turn contribute to the developmental and functional deficits in the Ts1Cje brain. Disomic DEGs that were clustered with the 3 interferon receptors include activin receptor IIB (*Acvr2b*), caspase 3 (*Casp3*), collagen, type XX, alpha 1 (*Col20a1*), ectodysplasin A2 isoform receptor (*Eda2r*), epidermal growth factor receptor (*Egfr*), c-fos induced growth factor (*Figf*), growth differentiation factor 5 (*Gdf5*), histocompatibility 2, K1, K region (*H2-K1*), interleukin 17 receptor A (*Il17ra*), interferon regulatory factor 3 (*Irf3*), interferon regulatory factor 7 (*Irf7*), inositol 1,4,5-triphosphate receptor 3 (*Itpr3*), lymphocyte cytosolic protein 2 (*Lcp2*), leptin receptor (*Lepr*), nuclear factor of activated T-cells, cytoplasmic, calcineurin-dependent 4 (*Nfatc4*), regulator of G-protein signaling 13 (*Rgs13*), signal transducer and activator of transcription 1 (*Stat1*) and Tnf receptor-associated factor 6 (*Traf6*). We consider these as important candidates for further analysis to understand the neuropathology of DS. We propose that differential regulation of these disomic genes will lead to a number of further cascades of low-level gene dysregulation within the Ts1Cje brain. For example, we found *Egfr* to be interconnected in various dysregulated molecular pathways represented by different functional clusters including the calcium signaling pathway, neuroactive ligand-receptor interaction and the MAPK signaling pathway, as well as pathways in cancers such as pancreatic and colorectal cancers, which involve focal adhesion and regulation of actin cytoskeleton (Figure 3).

We were also interested to elucidate all potential molecular pathways represented by the 18 DEGs that were common to all brain regions analysed throughout development (*Atp5o, Brwd1, Chaf1b, Cryzl1, Dnah11, Donson*, *Dopey2, Erdr1, Ifnar1, Ifnar2, Itgb8, Itsn1, Morc3, Mrps6, Pigp, Psmg1, Tmem50b and Ttc3*). Functional clustering analysis of these genes showed that interferon-related pathways were enriched, which was mainly attributed to the presence of *Ifnar1* and *Ifnar2*. Combining our functional analyses, our data suggest that interferon-related pathways are globally dysregulated and therefore important in causing neurological deficits within the Ts1Cje mouse brain.

### RT-qPCR validation of selected DEGs

**Figure 4:**
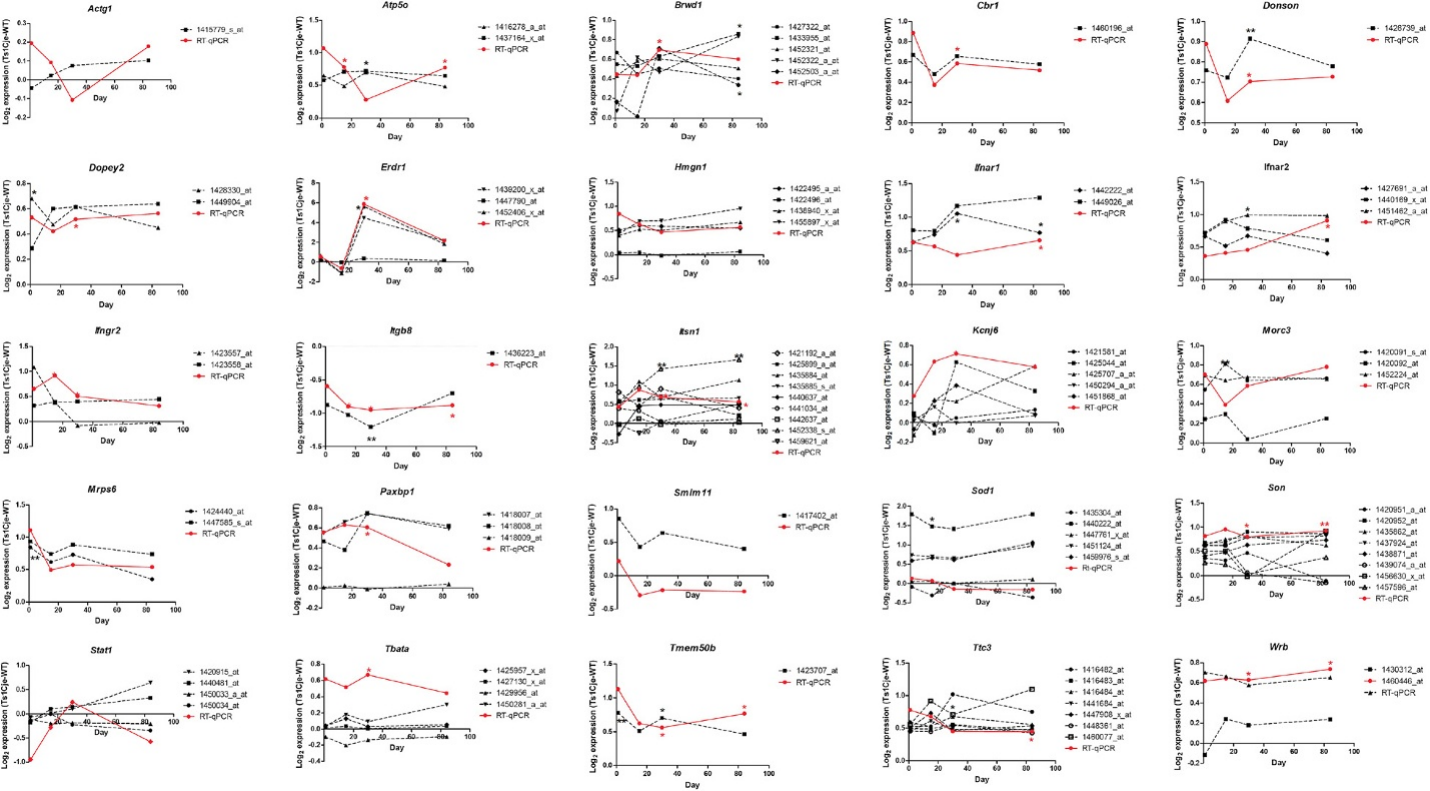
**RT-qPCR validation of selected DEGs in the cerebral cortex.** Red lines or asterisks denote RT-qPCR data whereas black lines or asterisks denote microarray data. *p < 0.05, **p < 0.01 and ***p < 0.001 based on Empirical Bayes t-statistic test.

**Figure 5:**
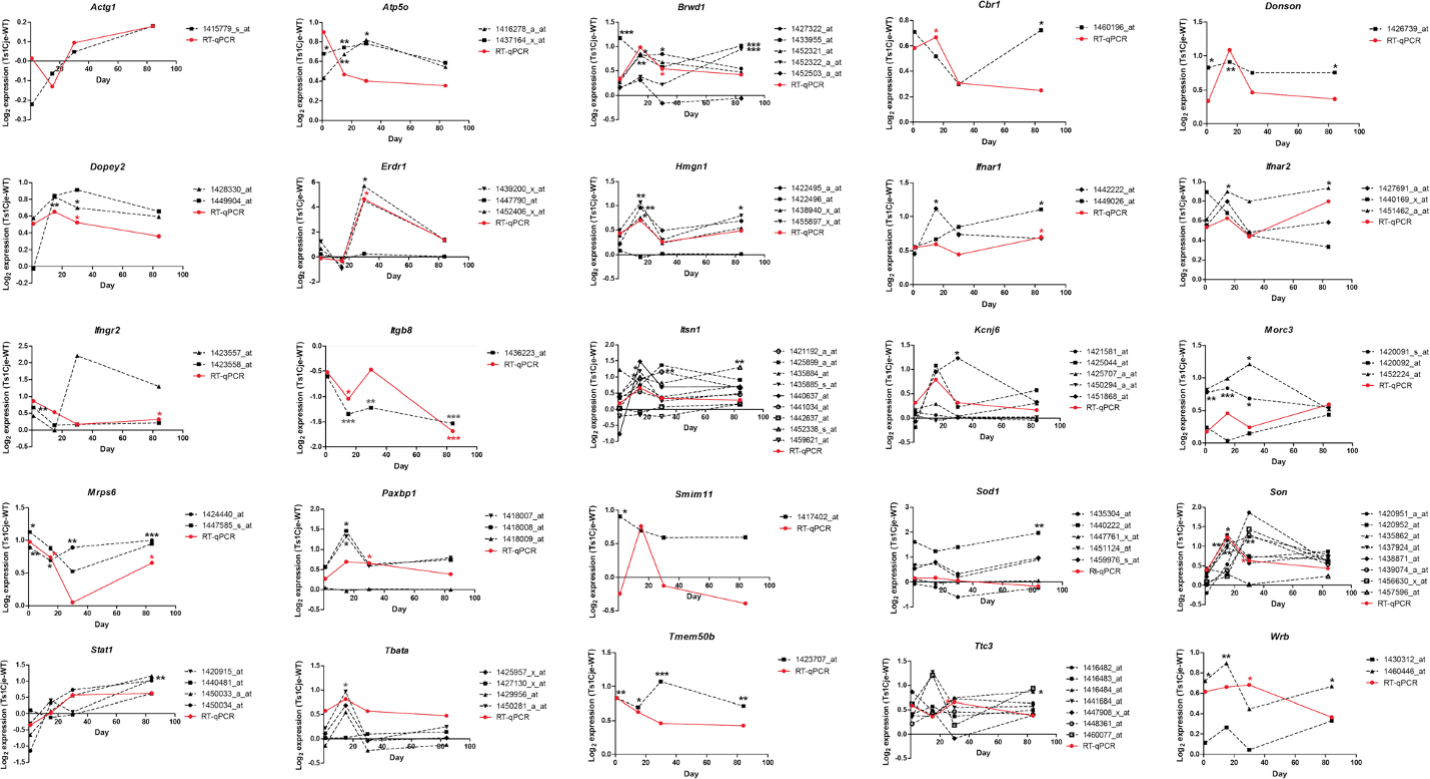
**RT-qPCR validation of selected DEGs in the cerebellum.** Red lines or asterisks denote RT-qPCR data whereas black lines or asterisks denote microarray data. *p < 0.05, **p < 0.01 and ***p < 0.001 based on Empirical Bayes t-statistic test

**Figure 6:**
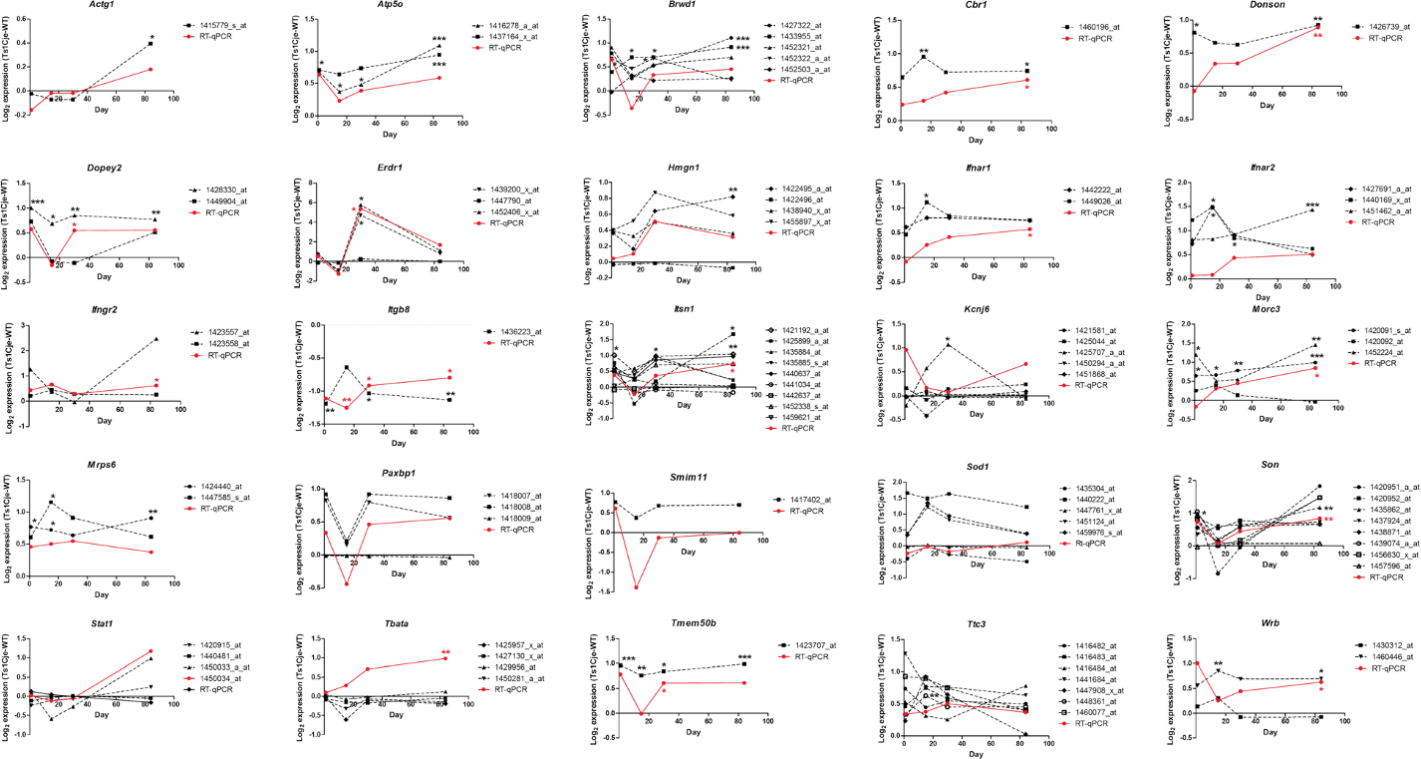
**RT-qPCR validation of selected DEGs in the hippocampus.** Red lines or asterisks denote RT-qPCR data whereas black lines or asterisks denote microarray data. *p < 0.05, **p < 0.01 and ***p < 0.001 based on Empirical Bayes t-statistic test.

RT-qPCR was used to validate the DEGs identified in the microarray comparisons. We focused on validating DEGs that were located within the triplicated MMU16 region, which were common to all brain regions analysed and those that were involved in interferon-related pathways. Twenty five genes (actin, gamma, cytoplasmic 1, (*Actg1*); *Atp5o*; *Brwd1*; *Cbr1*; *Donson*; *Dopey2*; *Erdr1*; high mobility group nucleosomal binding domain 1, (*Hmgn1*); *Ifnar1*; *Ifnar2*; *Ifngr2*; *Itgb8*; *Itsn1*; potassium inwardly-rectifying channel, subfamily J, member 6, (*Kcnj6*); *Morc3*; *Mrps6*; PAX3 and PAX7 binding protein 1, (*Paxbp1*); small integral membrane protein 11*, (Smim11)*; *Sod1*; Son cell proliferation protein, *(Son)*; *Stat1;* Thymus, brain and testes associated, *(Tbata)*; *Tmem50b*; *Ttc3* and tryptophan rich basic protei*n,* (*Wrb*)) were analysed by RT-qPCR using the same RNA that was used for the microarray analyses, which consisted of triplicate samples from Ts1Cje and disomic cerebral cortex tissues (Figure 4), cerebellum (Figure 5) and hippocampus (Figure 6) from the 4 postnatal stages (P1.5, P15, P30 and P84). The expression profile of a gene was considered to be validated when both microarray and RT-qPCR data showed a consistent directional change with fold differences ≥ 1.50 or ≤ 0.67. The microarray data include many genes that are represented by multiple probe-sets. For this analysis, only probe-sets that were considered to be statistically significant for each DEG were included. Eight of the selected DEGs were validated at various development time points in the cerebral cortex (*Brwd1*, *Donson*, *Erdr1*, *Ifnar1*, *Itgb8*, *Itsn1*, *Mrps6* and *Tmem50b*), 18 DEGs were validated in the cerebellum (*Atp5o*, *Brwd1*, *Donson*, *Dopey2*, *Erdr1*, *Hmgn1*, *Ifnar1*, *Ifnar2*, *Ifngr2*, *Itgb8*, *Itsn1*, *Mrps6*, *Paxbp1*, *Son*, *Stat1*, *Tbata, Tmem50b* and *Wrb*) and 11 DEGs were validated in the hippocampus (*Atp5o*, *Brwd1*, *Cbr1*, *Donson*, *Erdr1*, *Itgb8*, *Itsn1*, *Morc3*, *Son*, *Tmem50b* and *Wrb*). Detailed expression profiles for all 25 DEGs are summarized in Table 3.

**Table 3:** **Summary of spatiotemporal RT-qPCR validations of 25 selected DEGs**

Official symbol	Full gene name (ID)	Probe set ID	Log _2_ expression of Ts1Cje normalized against disomic littermates							
			Microarray analysis				RT-qPCR analysis			
			P1	P15	P30	P84	P1	P15	P30	P84
**Cerebral Cortex**										
*Atp5o*	ATP synthase, H+ transporting, mitochondrial F1 complex, O subunit	1437164_x_at	0.58	0.71	0.72*	0.64	1.07	0.78*	0.28	0.77*
*Brwd1*	Bromodomain and WD repeat domain containing 1	1433955_at	0.55	0.53	0.63	0.86*	0.44	0.44	0.70*	0.6
		1452322_a_at	0.07	0.62	0.47	0.83*				
*Donson*	Downstream neighbor of SON	1426739_at	0.76	0.72	0.91**	0.78	0.89	0.61	0.70*	0.73
*Dopey2*	Dopey family member 2	1428330_at	0.68*	0.47	0.61	0.45	0.53	0.42	0.52*	0.56
*Erdr1*	Erythroid differentiation regulator 1	1452406_x_at	0.61	-1.14	5.61*	1.82	0.54	-0.64	5.85*	2.16
*Ifnar1*	Interferon (alpha and beta) receptor 1	1449026_at	0.8	0.8	1.17*	1.29*	0.63	0.57	0.44	0.66*
*Ifnar2*	Interferon (alpha and beta) receptor 2	1451462_a_at	0.7	0.9	0.99*	0.98	0.36	0.41	0.45	0.91*
*Itgb8*	Integrin beta 8	1436223_at	-0.88	-1.02	-1.21**	-0.7	-0.59	-0.90*	-0.95*	-0.88*
*Itsn1*	Intersectin 1 (SH3 domain protein 1A)	1452338_s_at	0.58	0.96	1.42**	1.66**	0.43	0.88	0.69*	0.57*
*Morc3*	Microrchidia 3	1420091_s_at	0.55	0.81**	0.64	0.66	0.7	0.39	0.59	0.78
*Mrps6*	Mitochondrial ribosomal protein S6	1424440_at	0.84**	0.62	0.73	0.35	1.11	0.49	0.57	0.54
*Sod1*	Superoxide dismutase 1, soluble	1440222_at	1.79*	1.47	1.41	1.79	0.13	0.07	-0.15	-0.16
*Tmem50b*	*Transmembrane protein 50B*	1423707_at	0.78**	0.51	0.70*	0.46	1.12	0.62	0.56*	0.77*
*Ttc3*	Tetratricopeptide repeat domain 3	1448361_at	0.45	0.45	0.54*	0.43	0.78	0.68	0.45	0.45*
**Cerebellum**										
*Atp5o*	ATP synthase, H+ transporting, mitochondrial F1 complex, O subunit	1416278_a_at	0.43	0.67**	0.82*	0.54	0.90	0.47	0.40	0.36
		1437164_x_at	0.68*	0.74**	0.78	0.59				
*Brwd1*	Bromodomain and WD repeat domain containing 1	1427322_at	0.32	0.81*	0.84*	0.54	0.33	0.98	0.54*	0.42
		1433955_at	1.17***	0.81**	0.57	1.01***				
		1452322_a_at	0.14	0.37	0.22	0.94***				
*Cbr1*	Carbonyl reductase 1	1460196_at	0.71	0.52	0.30	0.72*	0.58	0.67*	0.31	0.25
*Donson*	Downstream neighbor of SON	1426739_at	0.82*	0.91**	0.75	0.75*	0.34	1.09	0.46	0.37
*Dopey2*	Dopey family member 2	1428330_at	0.58	0.83**	0.70*	0.59	0.50	0.65	0.52*	0.36
*Erdr1*	Erythroid differentiation regulator 1	1452406_x_at	0.67	-0.73	5.69*	1.34	-0.11	-0.31	4.63*	1.36
*Hmgn1*	High mobility group nucleosomal binding domain 1	1422495_a_at	0.22	0.96**	0.49	0.68*	0.43	0.69	0.26	0.48
		1438940_x_at	0.40	0.76*	0.24	0.54				
		1455897_x_at	0.49	1.07**	0.30	0.80				
*Ifnar1*	Interferon (alpha and beta) receptor 1	1442222_at	0.45	1.12*	0.73	0.68	0.55	0.60	0.44	0.70*
		1449026_at	0.55	0.67	0.85	1.10*				
*Ifnar2*	Interferon (alpha and beta) receptor 2	1451462_a_at	0.61	0.90*	0.80	0.93*	0.53	0.62	0.44	0.80
*Ifngr2*	Interferon gamma receptor 2	1423558_at	0.67**	0.14	0.17	0.21	0.86	0.53	0.17	0.32*
*Itgb8*	Integrin beta 8	1436223_at	-0.61	-1.35***	-1.22**	-1.54***	-0.52	-1.05*	-0.47	-1.69***
*Itsn1*	Intersectin 1 (SH3 domain protein 1A)	1421192_a_at	0.03	0.94*	1.16**	0.66	0.19	0.65	0.34	0.29
		1435885_s_at	0.46	1.18*	0.25	0.50				
		1452338_s_at	0.44	0.99	0.80	1.30**				
*Kcnj6*	Potassium inwardly-rectifying channel, subfamily J, member 6	1451868_at	-0.07	0.95	1.23*	0.33	0.31	0.79	0.32	0.16
*Morc3*	Microrchidia 3	1420091_s_at	0.78**	0.84***	0.69*	0.55	0.17	0.46	0.24	0.59
		1452224_at	0.82	0.99	1.21*	0.52				
*Mrps6*	Mitochondrial ribosomal protein S6	1424440_at	0.89**	0.70*	0.89**	1.00***	0.97	0.74*	0.05	0.66*
		1447585_s_at	1.13*	0.88	0.53	0.94				
*Paxbp1*	PAX3 and PAX7 binding protein 1	1418007_at	0.55	1.32*	0.59	0.80	0.27	0.69	0.66*	0.38
		1418008_at	0.56	1.45*	0.63	0.75				
*Smim11*	Small integral membrane protein 11	1417402_at	0.90*	0.69	0.59	0.59	-0.24	0.76	-0.13	-0.39
*Sod1*	Superoxide dismutase 1, soluble	1440222_at	1.60	1.24	1.40	1.96**	0.15	0.17	0.06	-0.17
*Son*	Son cell proliferation protein	1420952_at	0.36	1.28*	0.71	0.87	0.39	1.23	0.63*	0.44
		1435862_at	0.35	1.04**	1.24**	0.78				
		1437924_at	0.28	0.96**	0.75	0.67				
*Stat1*	Signal transducer and activator of transcription 1	1420915_at	-0.30	0.40	0.06	1.03**	-0.34	0.03	0.57	0.62
*Tbata*	Thymus, brain and testes associated	1450281_a_at	0.22	0.97*	-0.03	0.25	0.57	0.81*	0.57	0.47
*Tmem50b*	Transmembrane protein 50B	1423707_at	0.83**	0.69*	1.07***	0.71**	0.83	0.62	0.46	0.42
*Ttc3*	Tetratricopeptide repeat domain 3	1416484_at	0.60	0.39	0.74	0.87*	0.59	0.36	0.66*	0.38
*Wrb*	Tryptophan rich basic protein	1460446_at	0.72*	0.90**	0.45	0.67*	0.61	0.66	0.68*	0.37
**Hippocampus**										
*Actg1*	Actin, gamma, cytoplasmic 1	1415779_s_at	-0.02	-0.07	-0.07	0.39*	-0.16	-0.02	-0.02	0.18
*Atp5o*	ATP synthase, H+ transporting, mitochondrial F1 complex, O subunit	1416278_a_at	0.68	0.37	0.48	1.09***	0.64	0.23	0.39	0.59
		1437164_x_at	0.71*	0.64*	0.74*	0.94***				
*Brwd1*	Bromodomain and WD repeat domain containing 1	1427322_at	0.78	0.26	0.52	1.11***	0.66	-0.36	0.33	0.45
		1433955_at	0.39	0.70*	0.70*	0.91***				
		1452322_a_at	0.67*	0.46	0.68*	0.22				
*Cbr1*	Carbonyl reductase 1	1460196_at	0.64	0.95**	0.72	0.74*	0.24	0.30	0.42	0.61*
*Donson*	Downstream neighbor of SON	1426739_at	0.81*	0.65	0.63	0.92**	-0.08	0.34	0.34	0.89**
*Dopey2*	Dopey family member 2	1428330_at	1.00***	0.68*	0.85**	0.77**	0.57	-0.16	0.55*	0.55
*Erdr1*	Erythroid differentiation regulator 1	1439200_x_at	0.68	-0.94	4.72*	0.80	0.51	-1.27	5.36*	1.68
		1452406_x_at	0.80	-1.12	5.77*	1.10				
*Hmgn1*	High mobility group nucleosomal binding domain 1	1422495_a_at	0.36	0.16	0.64	0.82**	0.04	0.10	0.51	0.31
*Ifnar1*	Interferon (alpha and beta) receptor 1	1449026_at	0.46	1.12*	0.84	0.75	-0.08	0.25	0.41	0.57*
*Ifnar2*	Interferon (alpha and beta) receptor 2	1427691_a_at	0.73	1.49*	0.91	0.50	0.07	0.08	0.43	0.50
		1440169_x_at	1.22	1.49*	0.84	0.62				
		1451462_a_at	0.81	0.83	0.92*	1.43***				
*Itgb8*	Integrin beta 8	1436223_at	-1.19**	-0.64	-1.04*	-1.14**	-1.11	-1.26**	-0.92*	-0.80*
*Itsn1*	Intersectin 1 (SH3 domain protein 1A)	1421192_a_at	1.01*	0.48	0.97*	1.04**	0.38	-0.23	0.36	0.73*
		1425899_a_at	0.76	-0.54	0.18	1.67*				
*Kcnj6*	Potassium inwardly-rectifying channel, subfamily J, member 6	1425707_a_at	-0.21	0.57	1.07*	-0.06	0.96	0.17	0.07	0.66
*Morc3*	Microrchidia 3	1420091_s_at	0.64*	0.66*	0.78**	0.99***	-0.16	0.31	0.45	0.85*
		1452224_at	1.20*	0.51	0.55	1.45**				
*Mrps6*	Mitochondrial ribosomal protein S6	1424440_at	0.77*	0.72*	0.64	0.91**	0.46	0.50	0.55	0.38
		1447585_s_at	0.61	1.15*	0.91	0.61				
*Son*	Son cell proliferation protein	1435862_at	0.99*	0.20	0.58	1.17**	0.73	0.07	0.44	0.84**
*Tmem50b*	Transmembrane protein 50B	1423707_at	0.96***	0.76**	0.84*	0.99***	0.78	-0.01	0.61*	0.61
*Ttc3*	Tetratricopeptide repeat domain 3	1448361_at	0.33	0.63**	0.45	0.46	0.34	0.37	0.50	0.37
*Wrb*	Tryptophan rich basic protein	1460446_at	0.56	0.85**	0.69	0.70*	1.00	0.26	0.44	0.63*

All selected DEGs are trisomic genes located on chromosome 16 except for *Erdr1* (a disomic gene located on chromosome X), *Itgb8* (a monosomic gene located on chromosome 12), *Sod1* (a trisomic gene located on chromosome 16, although one of the copies is non-functional due to truncation), and *Stat1* (a disomic gene located at chromosome 1).

*p<0.05, **p<0.01 and ***p<0.001 based on Empirical Bayes t-statistic test.

### Western blotting

**Figure 7:**
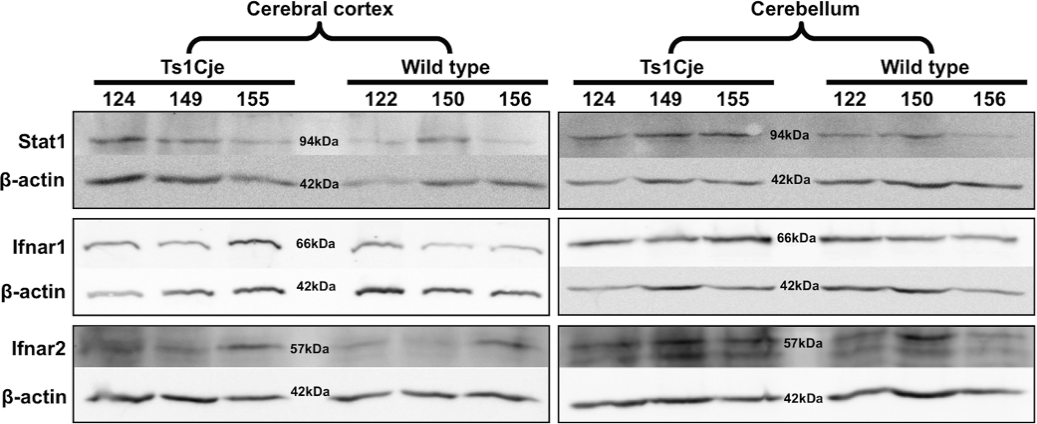
**Western blotting analysis of Ifnar1 (66 kDa), Ifnar2 (55 kDa) and Stat1 (91 kDa) in the cerebral cortex and cerebellum of adult (P84) Ts1Cje and wild type littermates.** Each band represents each Ts1Cje or wild type mouse in the respective brain region.

Both microarray and RT-qPCR analyses demonstrated significant differences in *Ifnar1, Ifnar2* and *Stat1* expression levels in the P84 cerebral cortex and cerebellum. To evaluate the effect of mRNA levels on protein synthesis, we measured the expression level of these proteins in the cerebral cortex and cerebellum lysates prepared from P84 Ts1Cje and wild type mice (Figure 7). Based on the pixelation analysis of the bands, Ifnar1 and Stat1 were found to be significantly (*p* ≤ 0.05) over-expressed in the Ts1Cje cerebellum as compared to wild type with 2.69- and 4.93-fold increases, respectively. In Ts1Cje cerebral cortices, we observed 1.55- and 1.73-fold upregulation of Ifnar1 and Ifnar2 expression, respectively, when compared to wild type. However, none of them were statistically significant based on pixelation analysis (see Additional file 4).

## Discussion

This study aimed to identify disruptions in molecular pathways caused by the partial trisomy of mouse chromosome 16 (MMU16) harbored by Ts1Cje mice, which results in neuropathology similar to that observed in people with DS. We provide the most comprehensive molecular expression catalogue for the Ts1Cje developing postnatal brain to date. Previous studies have focused on single brain regions or the whole brain at limited developmental stages [23, 29, 31–34]. We completed a stringent microarray analysis throughout postnatal development (P1.5, P15, P30 and P84) of the cerebral cortex, cerebellum and hippocampus of Ts1Cje versus disomic littermates. The majority of the trisomic probe-sets have a 0.5-fold increase in expression in Ts1Cje mice as compared to disomic controls. Our data are in agreement with previously reported microarray analysis involving Ts1Cje and disomic littermate control primary neural stem and progenitor cells [29] and Ts1Cje P0 mouse whole brains [33] or the cerebellum [32], which demonstrated a dosage-dependent over-expression of genes on the triplicated segment of MMU16.

According to the spatial analysis, the number of DEGs identified in the cerebellum and hippocampus was consistently higher than in the cerebral cortex at all time points. It is widely accepted that the cerebral cortex is the most highly developed part of the brain, and is responsible for the majority of information processing and higher cognitive functions, as well as being the most recent addition in evolutionary terms. We hypothesise that the smaller number of DEGs in this region throughout post-natal development represents the higher level of genetic control required for the cerebral cortex to function at a level that allows survival. Further evidence that supports this theory includes a meta-analysis [41] demonstrating that the human cortex has a reproducible genomic aging pattern whilst the cerebellum does not. This reproducibility reflects a higher level of gene expression control in the cortex compared to the cerebellum even through the degenerative process of aging to maintain a certain level of function.

The Ts1Cje mouse model contained a partial monosomy of MMU12 following partial translocation of MMU16 onto this site. An ~2 MB segment of the telomeric end of MMU12 is deleted [23], and consequently seven genes were deleted (*Abcb5, Dnah11, Itgb8, Macc1, Sp4, Sp8,* and *Tmem196*) [42]. Our data showed that dynein axonemal heavy chain 11 (*Dnah11*) is significantly up-regulated in all three brain regions and four postnatal developmental time points with a log_2_ expression ratio that ranged from 5.4 to 7.7. This over-expression of *Dnah11* is consistent with previously reported cerebellum microarray expression results [23] and this over-expression is probably specific to the Ts1Cje mouse model [23, 33] since similar over-expression in DS patients or the Ts65Dn mouse model has not been observed [43–46]. Over-expression of the *Dnah11* gene is likely caused by the position effect of an upstream regulatory element following translocation onto MMU12 in the Ts1Cje genome. In our study, the expression levels of *Sp8* and *Itgb8* are down-regulated (Additional file 2: Table S2) as they are monosomic in Ts1Cje [42]. *Sp8,* trans-acting transcription factor 8, is important for patterning in the developing telencephalon, specification of neuronal populations [47] and also neuromesodermal stem cell maintenance and differentiation via Wnt3a [48]. Meanwhile, *Itgb8,* Intergrin beta 8, is crucial for neurogenesis and neurovascular homeostasis regulation [49]. This down-regulation of *Sp8* and *Itgb8* may affect DS neuropathology features to a certain extent in the Ts1Cje mouse brain. The remaining four monosomic genes in Ts1Cje mice [(ATP-binding cassette, sub-family B (MDR/TAP), member 5, (*Abcb5*); metastasis associated in colon cancer 1, (*Macc1*); trans-acting transcription factor 4, *(Sp4)* and transmembrane protein 196 Mus musculus, (*Tmem196*)] were not found to be dysregulated in our data.

Our data are also in agreement with a previously reported meta-analysis that was performed on DS patient tissues, cell lines and mouse models at different developmental stages [50]. Fifteen of the top 30 DS trisomic genes with direct dosage effects reported in the meta-analysis report [50] were also selected as DEGs in our analysis [(*Cbr1; carbonyl reductase, (Cbr3); Donson;* Down syndrome critical region gene 3*, (Dscr3);* E26 avian leukemia oncogene 2, 3' domain, (*Ets2);* phosphoribosylglycinamide formyltransferase, *(Gart); Ifnar2; Ifngr2; Psmg1;* regulators of calcineurin 1, *(Rcan1); Son; synaptojanin 1, (Synj1); Tmem50b, Ttc3* and *Wrb*)]. The expression of dual-specificity tyrosine-(Y)-phosphorylation regulated kinase 1a (*Dyrk1a*), a well-studied gene in DS individuals and mouse models, has been found to be inconsistent across various expression profiling studies involving the brain of Ts1Cje mice. *Dyrk1a* was not differentially regulated in our dataset and our finding is in agreement with two other studies on the embryonic Ts1Cje neurosphere [34] and early postnatal Ts1Cje whole brains [33], but this result is in contrast to those of Laffaire et al. [23], who observed *Dyrk1a* over-expression in the cerebellum of early postnatal Ts1Cje mice. According to our dataset, *Rcan1*, which is located in the Down syndrome critical region (DSCR), was over-expressed in P1 cerebral cortex and P15 hippocampus of Ts1Cje mice. *Rcan1-*null mice demonstrated deficits in spatial learning and memory, implicating its role in late-phase long-term potentiation and memory formation [51]. In addition, RCAN1-1S over-expression in the hippocampal neuronal cell line HT22 cell line resulted in hyperphosphorylation of tau [52], which positions *Rcan1* as an important candidate for further investigation in DS-related Alzheimer’s disease features.

Functional clustering of various DEGs based on DAVID ontologies highlighted a global dysregulation of interferon-related molecular networks in all brain regions attributed mainly to the dysregulated expression of the trisomic genes *Ifnar1* and *Ifnar2*. These genes code for IFN beta-receptor subunits 1 and 2, respectively. However, *Ifngr2*, which encodes one of the two subunits of the IFN gamma receptor, was differentially upregulated in the cerebellum only. A role for all 3 interferon receptors and their dysregulation has been described in mouse models of DS. For example, mouse fetuses that are trisomic for MMU16 (Ts16), which includes the interferon alpha and gamma receptor genes, showed upon subsequent knockout of these genes improved growth when compared to Ts16 fetuses and generated cortical neurons with similar viability to their euploid counterparts [53]. In the present study, upregulation of these receptors suggests that the Ts1Cje mouse would have a lower response threshold or hyperresponsiveness to interferons or cytokines that would result in activation of downstream intracellular signaling pathways contributing to the observed neuropathology, particularly in the cerebellum.

In addition to *Ifnar1*, *Ifnar2* and *Ifngr2*, our analysis showed that other Jak-Stat- associated genes such as *Stat1* (P84), *Lepr* (P1) and two interferon response factor genes, *Irf3* (P15) and *Irf7* (P84), were upregulated in the Ts1Cje cerebellum. Irf3 and Irf7 have been shown to induce type 1 interferons, which subsequently stimulate Jak-Stat signal transduction pathways leading to upregulated transcription of various interferon-stimulated genes [54–56]. Leptin and its receptor, Lepr, have been shown to be involved in leptin-dependent adult hippocampal neurogenesis [57] and mediated neuroprotection of dopaminergic cells via activation of Jak-Stat, mitogen-activated protein kinases (MEK)/extracellular signal-regulated kinases (ERK) and growth factor receptor-bound protein 2 (GRB2) signaling pathways in a mouse model of Parkinson’s disease [58]. The role of the Jak-Stat signaling pathway in the brain, however, is unclear. Jak-Stat signaling has recently been implicated in neurogenesis/cell-fate determination [59, 60], astrogliogenesis [61, 62] and synaptic plasticity [63, 64] within the nervous system of rats and fruit flies, but not specifically in the development and progression of neuropathology in mouse models or individuals with DS. Elevation of STAT1 activities has been shown to promote astrogliogenesis during the neurogenic phase of development [61]. We have previously demonstrated that Ts1Cje mice have a number of defects in adult neurogenesis, including a severe reduction in the numbers of neurons produced and an increased number of astrocytes [29]. Our current protein analysis further confirmed the over-expression of Ifnar1 and Stat1 in the cerebellum of adult (P84) Ts1Cje mice as compared to their wild type littermates. Therefore, we hypothesize that over-activation of Jak-Stat signal transduction, which is due to the increased sensitivity towards interferons via over-expression of interferon receptor, may lead to a preference for the glial-fated path in Ts1Cje neural precursors that contributes to the neuropathology observed in Ts1Cje mice. The role of the trisomic genes *Ifnar1*, *Ifnar2* and *Ifngr2* and the disomic gene *Lepr* in upregulation of *Stat1*, *Irf3* and *Irf7* and subsequent activation of Jak-Stat signaling in the Ts1Cje mouse brain, particularly the cerebellum, remains elusive and warrants further investigation.

From the list of validated trisomic DEGs, *Brwd1*, *Donson*, *Tmem50b* and *Itsn1* were upregulated in all brain regions, which concurs with previous studies [65–72]. Both *Brwd1* and *Donson* are not well studied and have not been associated with the progression and development of neuropathology in DS. *Brwd1* encodes a nuclear protein that plays a role in transcriptional regulation related to diverse biological functions [65, 66]. *Donson*, on the other hand, encodes a protein of unknown function. Fusion transcripts that are encoded by exons from *Donson* and another trisomic DEG, *Atp5o*, have been reported but their role/function also remains unknown [67]. *Tmem50b* encodes an intracellular membrane protein expressed mainly in the endoplasmic reticulum and Golgi apparatus of the rodent brain [68]. At the subcellular level, *Tmem50b* is expressed in rat and mouse glial fibrillary acidic protein (GFAP)-positive cells and to a lesser degree in neuronal microtubule-associated protein 2 (MAP2)- or beta-tubulin II-positive cells *in vitro*, suggesting a role for this gene in astroglial cell development or function. Upregulation of *ITSN1* has been demonstrated previously in the prosencephalon of DS fetuses compared with controls [69]. *Itsn1* is also expressed in both proliferating and differentiating neurons in the mouse brain [69] and has been shown to regulate endocytosis events probably via the formation of clathrin-coated vesicles, which are important for recycling synaptic vesicles [70]. Endocytosis anomalies such as enlarged endosomes in neurons were identified as an early neuropathological feature in the brain of Ts65Dn mice and individuals with DS and Alzheimer’s disease [71, 72]. Over-expressed *Itsn1* and amyloid beta (A4) precursor protein (*App*) may contribute to the early development of Alzheimer’s disease in DS individuals by accelerating beta amyloid and neurofibrillary tangle accumulation via increased endocytosis activity in neurons.

Our microarray data demonstrate that many other trisomic DEGs such as *Atp5o*, *Cbr1*, *Dopey2*, *Erdr1*, *Hmgn1*, *Morc3*, *Mrps6*, *Son* and *Wrb*, are upregulated in Ts1Cje mouse brain regions. The molecular and cellular functions of these DEGs have not been comprehensively characterized in the brain and therefore their potential roles in the onset and progression of neuropathology observed in DS remain poorly understood. Of these DEGs, the expression profiles of *Cbr1*, *Dopey2*, *Erdr1*, *Hmgn1* and *Mrps6* are in agreement with previous studies of DS mouse models [31, 32, 73–75]. The chromatin-binding protein *Hmgn1* is a negative regulator of methyl CpG-binding protein 2 (*MeCP2*) expression via chromatin structure changes and histone modification in the *MeCP2* promoter [76]. As *MeCP2* has widespread effects on gene expression, especially in neurological disease such as Rett syndrome [77], over-expressed *Hmgn1* will down-regulate *MeCP2* expression, which may cause disruption in terms of downstream gene expression that is necessary for normal brain development. *Dopey2* has been proposed as a candidate gene that is responsible for mental retardation in DS individuals because its expression was found in brain regions that are involved in learning and memory processes [75, 78–80]. Transgenic mice over-expressing Dopey2 demonstrated increased density of cortical cells suggesting that this protein may play an important role in brain morphogenesis and therefore may contribute to neuropathology of DS when over-expressed [78, 80]. These under-characterised DEGs are important candidates that should be investigated further to understand various neuropathological features of DS.

## Conclusion

Our study aimed to define the disrupted molecular pathways caused by partial triplication of MMU16 during postnatal brain development in the Ts1Cje mouse model of DS. Global analysis of transcriptomes from different regions of the Ts1Cje brain supported a gene-dosage effect of the majority of the trisomic genes that led to the disruption of the disomic genome. Interferon-related pathways were identified as the most significantly dysregulated molecular networks and these changes were attributed mainly to the upregulation of the interferon receptors, which are encoded by the trisomic genes *Ifnar1*, *Ifnar2* and *Ifngr2*. Upregulation of Ifnar1 and Stat1 proteins in the adult Ts1Cje cerebral cortex and cerebellum suggests that interferon receptor over-expression may lead to over-stimulation of Jak-Stat signaling pathway. The role of interferon-mediated activation or inhibition of signal transduction has been well-characterized in various biological processes and disease models, including DS, but information pertaining to its role in the development and function in the Ts1Cje or DS brain remains scarce and warrants further investigation.

## Electronic supplementary material

Additional file 1: Table S1: List of primers and UPL probes used for RT-qPCR validations. (DOCX 18 KB)

Additional file 2: Table S2: List of differentially expressed genes (DEGs) identified based on spatiotemporal analysis of various brain regions and developmental timepoints of Ts1Cje. (XLSX 164 KB)

Additional file 3: Table S3: List of significant annotation clusters based on the analysis of functional ontologies using DAVID tools. (DOCX 35 KB)

Additional file 4: Figure S4: Western blotting analysis for Stat1, Ifnar1 and Ifnar2 protein expression in the P84 cerebral cortex and cerebellum of Ts1Cje and wild type littermates. **Table S4**: Pixelation analysis of Stat1, Ifnar1 and Ifnar2 bands detected on Western blots. (DOCX 320 KB)
