# Supplementary material for: Functional transcriptome analysis of the postnatal brain of the Ts1Cje mouse model for Down syndrome reveals global disruption of interferon-related molecular networks
Source: BMC Genomics. 2014 Jul 22;15(1):624. doi: 10.1186/1471-2164-15-624 (PMC4124147; doi:10.1186/1471-2164-15-624)
Supplement: Supplementary file 1 — Additional file 1: Table S1: List of primers and UPL probes used for RT-qPCR validations. (DOCX 18 KB) [file 12864_2014_6325_MOESM1_ESM.docx]

**Supplementary File 1**

**Table S1: List of primers and UPL probes used for RT-qPCR validations.**

| **Gene** | **Forward primers** | **GC%** | **Reverse primers** | **GC%** | **Amplicon size (nt)** | **UPL Probe (#)** | **Remarks** |
| --- | --- | --- | --- | --- | --- | --- | --- |
| Actg1 | cctagcacctagcacgatga | 55 | actcctgcttgctgatccac | 55 | 128 | 11 | ISP |
| Atp5o | atctgtggtcaggcccttt | 53 | tggtccagcttcttctcctt | 53 | 117 | 84 | ISP |
| Brwd1 | catgtcctcaaggtgtggag | 55 | caaacacttcatcagcatgtcc | 55 | 76 | 47 | ISP |
| Cbr1 | ccgagatgtctgcaaggag | 58 | ctcaccatgctggacacatt | 58 | 75 | 13 | ISP |
| Donson | tgttggcctgtctctggataa | 48 | agcactggcctctgactga | 48 | 111 | 1 | ISP |
| Dopey2 | agtgtgcaagcgctctctct | 55 | cggtaaaccatgtccaggag | 55 | 62 | 53 | ISP |
| Erdr1 | gacggagcgattctcacg | 61 | ggcatttctgtacgcagtca | 61 | 63 | 12 | NISP |
| Hmgn1 | atggagagacggaaaaccag | 50 | gacgtgatggatgcttagtcg | 50 | 76 | 110 | ISP |
| Ifnar1 | gtgcagtgtataagcaccacagt | 48 | ggacatagctcttgccttgg | 48 | 85 | 72 | ISP |
| Ifnar2 | tgagcaggatgcgttcac | 56 | tctctaggctcgcagacacc | 56 | 81 | 73 | ISP |
| Ifngr2 | tcctgtcacgaaacaacagc | 50 | acggaatcaggatgacttgc | 50 | 61 | 64 | ISP |
| Itgb8 | tccagccttggttcttgg | 56 | cacatttgcagagccacatc | 56 | 91 | 64 | ISP |
| Itsn1 | tgaatccagaagtcacgatgag | 45 | ctccagtctggctttcatcc | 45 | 74 | 20 | ISP |
| Kcnj6 | cctaccctgtccactacaagga | 55 | ctttccacatcctggtccat | 55 | 112 | 97 | ISP |
| Morc3 | cgtgcagcttgatgatgtg | 53 | cttctctatttccaacaactgaacc | 53 | 94 | 27 | ISP |
| Mrps6 | gctcccctacaggatctcg | 63 | agcataaaaatccaccaggaaa | 63 | 73 | 76 | ISP |
| Paxbp1 | gaaggatgaggctgacgttg | 55 | cccacatggtttcagctatca | 55 | 89 | 74 | ISP |
| Smim11 | aggaaaactgcaagcagagaa | 43 | tccacccaagctgacattta | 43 | 96 | 38 | ISP |
| Sod1 | caggacctcattttaatcctcac | 43 | cccaggtctccaacatgc | 43 | 76 | 49 | ISP |
| Son | gttaaaccgagctcaagaacg | 48 | ccagctgctcctgtgtcag | 48 | 107 | 99 | ISP |
| Stat1 | gcaggtgttgtcagatcgaa | 50 | tgtcgttctaccacgaagga | 50 | 72 | 101 | ISP |
| Tbata | ttcttatcccgctgtctgatg | 48 | agtttcttcctccaggtgtcag | 48 | 110 | 105 | ISP |
| Tmem50b | ccggactgaggttgatcg | 61 | catggagttctcattaaacaatgg | 61 | 126 | 38 | ISP |
| Ttc3 | aatggctctcgactcagtgac | 52 | gaggaactgaggatggatcg | 52 | 87 | 47 | ISP |
| Wrb | aagtattactccgtccctgtgg | 50 | ggtgattccaattccacctg | 50 | 105 | 104 | ISP |
| Pgk1 | tacctgctggctggatgg | 61 | cacagcctcggcatatttct | 50 | 65 | 108 | HKG |
| Psmb2 | gagggcagtggagcttctta | 55 | aggtgggcagattcaagatg | 50 | 71 | 25 | HKG |
| Hmbs | aaagttccccaacctggaat | 45 | ccaggacaatggcactgaat | 50 | 98 | 42 | HKG |

ISP = Intron-spanning primers; NISP = Non intron-spanning primers; HKG = Housekeeping genes
