# Supplementary material for: Functional transcriptome analysis of the postnatal brain of the Ts1Cje mouse model for Down syndrome reveals global disruption of interferon-related molecular networks
Source: BMC Genomics. 2014 Jul 22;15(1):624. doi: 10.1186/1471-2164-15-624 (PMC4124147; doi:10.1186/1471-2164-15-624)
Supplement: Supplementary file 3 — Additional file 3: Table S3: List of significant annotation clusters based on the analysis of functional ontologies using DAVID tools. (DOCX 35 KB) [file 12864_2014_6325_MOESM3_ESM.docx]

**Supplementary file 3**

**Table S3-1: List of significant annotation clusters based on the analysis of functional ontologies using DAVID tools on 317 selected DEGs.**

| **Enrichment Score: 1.78** | ***Ifnar1, Ifnar2, Stat1, Casp3, Cd70, H2-K1, Ifngr2, Lcp2, Nfatc4, Acvr2b, Figf, Eda2r, Egfr, Gdf5, Il17ra, Lepr, Traf6, Irf3, Irf7, Irf9, Col20a1, Itpr3, Rgs13*** | | |
| --- | --- | --- | --- |
|  |  |  |  |
| **Term** | **No. of DEG(s)** | ***p-*Value** | **DEG(s)** |
| IFN alpha signaling pathway | 4 | 0.000292 | *Ifnar1, Ifnar2, Irf9, Stat1* |
| Cytokine-cytokine receptor interaction | 11 | 0.006455 | *Acvr2b, Cd70, Figf, Eda2r, Egfr, Gdf5, Ifnar1, Ifnar2, Ifngr2, Il17ra, Lepr* |
| Natural killer cell mediated cytotoxicity | 7 | 0.016808 | *Casp3, H2-K1, Ifnar1, Ifnar2, Ifngr2, Lcp2, Nfatc4* |
| Toll-like receptor signaling pathway | 6 | 0.02453 | *Traf6, Ifnar1, Ifnar2, Irf3, Irf7, Stat1* |
| Jak-STAT signaling pathway | 6 | 0.099215 | *Ifnar1, Ifnar2, Ifngr2, Irf9, Lepr, Stat1* |
| Inflammation mediated by chemokine and cytokine signaling pathway | 8 | 0.285189 | *Col20a1, Itpr3, Ifnar1, Ifnar2, Ifngr2, Nfatc4, Rgs13, Stat1* |

| **Enrichment Score: 1.10** | ***Ddx58, Traf6, Ifnar1, Ifnar2, Ifngr2 ,Il33, Irf3, Irf7, Stat1*** | | |
| --- | --- | --- | --- |
|  |  |  |  |
| **Term** | **No. of DEG(s)** | ***p-*Value** | **DEG(s)** |
| Toll-like receptor signaling pathway | 6 | 0.02453 | *Traf6, Ifnar1, Ifnar2, Ifngr2, Irf3, Irf7, Stat1* |
| Cytosolic DNA-sensing pathway | 4 | 0.056229 | *Ddx58, Il33, Irf3, Irf7* |
| RIG-I-like receptor signaling pathway | 4 | 0.094577 | *Ddx58, Traf6, Irf3, Irf7* |
| Toll receptor signaling pathway | 3 | 0.316828 | *Traf6, Irf3, Irf7* |

| **Enrichment Score: 1.00** | ***Dtx1, Kat2b Lfng,Notch3*** | | |
| --- | --- | --- | --- |
|  |  |  |  |
| **Term** | **No. of DEG(s)** | ***p-*Value** | **DEG(s)** |
| Notch signaling pathway | 4 | 0.043040 | *Dtx1, Kat2b Lfng,Notch3* |

| **Enrichment Score: 0.95** | ***Avpr1a, Egfr, Lepr, Hrh1, Itpr3, Ntsr1, Tacr3, Trhr, Tnnc2*** | | |
| --- | --- | --- | --- |
|  |  |  |  |
| **Term** | **No. of DEG(s)** | ***p-*Value** | **DEG(s)** |
| Calcium signaling pathway | 8 | 0.031997 | *Avpr1a, Egfr, Hrh1, Itpr3, Ntsr1, Tacr3, Trhr, Tnnc2* |
| Neuroactive ligand-receptor interaction | 6 | 0.392874 | *Avpr1a, Hrh1, Lepr, Ntsr1, Tacr3, Trhr* |

| **Enrichment Score: 0.43** | ***Traf1, Traf6, Casp3, Egfr, Fdz2, Figf, Fgf3, Nfatc4, Pla2g12a, Ppm1a, Stat1*** | | |
| --- | --- | --- | --- |
|  |  |  |  |
| **Term** | **No. of DEG(s)** | ***p-*Value** | **DEG(s)** |
| MAPK signaling pathway | 7 | 0.316054 | *Traf6, Casp3, Egfr, Fgf3, Nfatc4, Pla2g12a, Ppm1a* |
| Pathways in cancer | 8 | 0.348787 | *Traf1, Traf6, Figf, Casp3, Egfr, Fgf3, Fzd2, Stat1* |
| Pancreatic cancer | 3 | 0.369888 | *Figf, Egfr, Stat1* |
| Colorectal cancer | 3 | 0.451387 | *Casp3, Egfr, Fzd2* |

| **Enrichment Score: 0.34** | ***Itgb8, Actg1, Tnnt2*** | | |
| --- | --- | --- | --- |
|  |  |  |  |
| **Term** | **No. of DEG(s)** | ***p-*Value** | **DEG(s)** |
| Hypertrophic cardiomyopathy (HCM) | 3 | 0.433069 | *Itgb8, Actg1, Tnnt2* |
| Dilated cardiomyopathy | 3 | 0.481174 |  |

| **Enrichment Score: 0.32** | ***Egfr, Fgf3, Figf, Iqgap3, Itgb8, Parvg, Actg1*** | | |
| --- | --- | --- | --- |
|  |  |  |  |
| **Term** | **No. of DEG(s)** | ***p-*Value** | **DEG(s)** |
| Focal adhesion | 5 | 0.454844 | *Figf, Egfr, Itgb8, Parvg, Actg1* |
| Regulation of actin cytoskeleton | 5 | 0.506072 | *Iqgap3, Egfr, Fgf3, Itgb8, Actg1* |

**Table S3-2: List of significant annotation clusters based on the analysis of functional ontologies using DAVID tools on DEGs identified in the cerebral cortex, hippocampus and cerebellum regardless of developmental stages.**

| **Brain region** | **Pathway** | ***p*-Value** | **No. of DEG(s)** | **DEG(s)** |
| --- | --- | --- | --- | --- |
| **Cerebral Cortex** | Cytokine-cytokine receptor interaction | 0.012047 | 3 | *Ifnar1, Ifnar2, Il17ra* |
|  | Hypertrophic cardiomyopathy (HCM) | 0.047548 | 2 | *Itgb8, Tnnt2* |
|  | Dilated cardiomyopathy |  |  |  |
| **Hippocampus** | Neuroactive ligand-receptor interaction | 0.002374 | 4 | *Avpr1a, Ntsr1, Tacr3, Trhr* |
|  | Calcium signaling pathway | 0.006175 |  |  |
| **Cerebellum** | Cytokine-cytokine receptor interaction | 6.89E-07 | 8 | *Acvr2b, Cd70, Eda2r, Egfr, Ifnar1, Ifnar2, Ifngr2, Lepr* |
|  | Jak-STAT signaling pathway | 4.62E-06 | 6 | *Ifnar1, Ifnar2, Ifngr2, Irf9, Lepr, Stat1* |
|  | Natural killer cell mediated cytotoxicity | 4.23E-04 | 5 | *Casp3, Ifnar1, Ifnar2, Ifngr2, Nfatc4* |
|  | Pathways in cancer | 1.16E-06 | 7 | *Traf1, Traf6, Casp3, Egfr, Fgf3, Fzd2, Stat1* |
|  | MAPK signaling pathway | 4.23E-04 | 5 | *Traf6, Casp3, Egfr, Fgf3, Nfatc4* |
|  | Colorectal cancer | 0.0112 | 3 | *Casp3, Egfr, Fzd2* |
|  | RIG-I-like receptor signaling pathway | 8.99E-04 | 4 | *Ddx58, Traf6, Irf3, Irf7* |
|  | Cytosolic DNA-sensing pathway | 0.0215 | 3 | *Ddx58, Irf3, Irf7* |

**Table S3-3: List of significant annotation clusters based on the analysis of functional ontologies using DAVID tools on DEGs identified in each developmental stage for the cerebral cortex.**

| **Time point** | **Pathway** | ***p*-Value** | **No. of gene** | **Genes** |
| --- | --- | --- | --- | --- |
| **P30** | Toll-like receptor signaling pathway | 0.080370 | 2 | *Ifnar1, Ifnar2* |
|  | Natural killer cell mediated cytotoxicity |  |  |  |
|  | Jak-STAT signaling pathway |  |  |  |

**Table S3-4: List of significant annotation clusters based on the analysis of functional ontologies using DAVID tools on DEGs identified in each developmental stage for the cerebellum.**

| **Time point** | **Pathway** | ***p*-Value** | **No. of gene** | **Genes** |
| --- | --- | --- | --- | --- |
| **P1** | MAPK signaling pathway | 9.63E-04 | 4 | *Traf6, Egfr, Fgf3, Nfatc4* |
|  | Pathways in cancer | 0.003159 | 4 | *Traf6, Egfr, Fgf3, Fzd2* |
| **P15** | Toll-like receptor signaling pathway | 0.006397 | 3 | *Ifnar1, Ifnar2, Irf3* |
| **P30** | Huntington's disease | 0.013333 | 2 | *Casp3, Atp5o* |
|  | Alzheimer's disease | 0.019955 |  |  |
|  | Parkinson's disease | 0.019955 |  |  |
| **P84** | Toll-like receptor signaling pathway | 0.001395 | 4 | *Ifnar1, Ifnar2, Irf7, Stat1* |
|  | Jak-STAT signaling pathway | 0.001395 | 4 | *Ifnar1, Ifnar2, Irf9, Stat1* |
|  | IFN alpha signaling pathway | 0.016449 |  |  |

**Table S3-5: List of significant annotation clusters based on the analysis of functional ontologies using DAVID tools on DEGs identified in each developmental stage for the hippocampus.**

| **Time point** | **Pathway** | ***p*-Value** | **No. of gene** | **Genes** |
| --- | --- | --- | --- | --- |
| **P15** | Calcium signaling pathway | 0.010483 | 3 | *Ntsr1, Tacr3, Trhr* |
|  | Neuroactive ligand-receptor interaction | 0.010483 |  |  |
| **P84** | Cell adhesion molecules (CAMs) | 0.011084 | 3 | *H2-K1, Itgb8, Jam2* |
|  | Steroid biosynthesis | 0.011084 | 3 | *Fdft1, Sqle, Sc4mol* |
|  | Focal adhesion | 0.02132 | 3 | *Itgb8, Parvg, Actg1* |
