# Supplementary material for: Functional transcriptome analysis of the postnatal brain of the Ts1Cje mouse model for Down syndrome reveals global disruption of interferon-related molecular networks
Source: BMC Genomics. 2014 Jul 22;15(1):624. doi: 10.1186/1471-2164-15-624 (PMC4124147; doi:10.1186/1471-2164-15-624)
Supplement: Supplementary file 4 — Additional file 4: Figure S4: Western blotting analysis for Stat1, Ifnar1 and Ifnar2 protein expression in the P84 cerebral cortex and cerebellum of Ts1Cje and wild type littermates. Table S4: Pixelation analysis of Stat1, Ifnar1 and Ifnar2 bands detected on Western blots. (DOCX 320 KB) [file 12864_2014_6325_MOESM4_ESM.docx]

**Supplementary File 4**


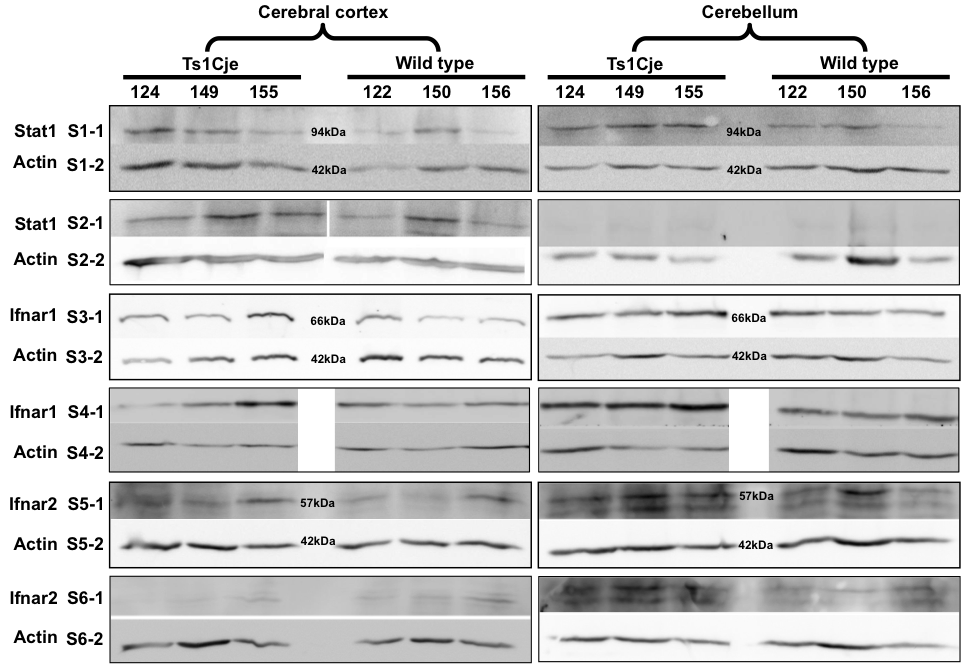


**Figure S4** Western blotting analysis for Stat1, Ifnar1 and Ifnar2 protein expression in the P84 cerebral cortex and cerebellum of Ts1Cje and wild type littermates. Technical replicates were performed for each protein, Stat1 (91kDa), Ifnar1 (66kDa), Ifnar2 (57kDa) and Actin (42kDa).

| **Label** | **Protein** | **Cerebral cortex** | | | | | | **Cerebellum** | | | | | |
| --- | --- | --- | --- | --- | --- | --- | --- | --- | --- | --- | --- | --- | --- |
|  |  | **Ts1Cje** | | | **Wild type** | | | **Ts1Cje** | | | **Wild type** | | |
|  |  | **Band 1** | **Band 2** | **Band 3** | **Band 4** | **Band 5** | **Band 6** | **Band 1** | **Band 2** | **Band 3** | **Band 4** | **Band 5** | **Band 6** |
| **S1-1** | **Stat1** | 7135 | 3079 | 1679 | 1308 | 3341 | 798 | 5812 | 9437 | 11005 | 2197 | 1803 | 1473 |
| **S1-2** | **Actin** | 11709 | 10723 | 6247 | 2666 | 5660 | 5789 | 7069 | 10799 | 7682 | 10756 | 12721 | 11001 |
| **Ratio A**  **= (S1-1/S1-2)** | | 0.6093 | 0.2872 | 0.2688 | 0.4908 | 0.5902 | 0.1378 | 0.8222 | 0.8738 | 1.433 | 0.2043 | 0.1417 | 0.1339 |
|  | | | | | | | | | | | | | |
| **S2-1** | **Stat1** | 3502 | 5736 | 3418 | 1608 | 3910 | 1960 | 353 | 312 | 623 | 499 | 1004 | 182 |
| **S2-2** | **Actin** | 12296 | 8674 | 6235 | 6590 | 6953 | 6211 | 4228 | 4742 | 2923 | 5445 | 12316 | 3356 |
| **Ratio B**  **= (S2-1/S2-2)** | | 0.2848 | 0.6612 | 0.5482 | 0.2440 | 0.5623 | 0.3156 | 0.0835 | 0.0659 | 0.2130 | 0.0916 | 0.0815 | 0.0542 |
|  | | | | | | | | | | | | | |
| **Average of A & B** | | 0.4471 | 0.4742 | 0.4085 | 0.3674 | 0.5762 | 0.2267 | 0.4529 | 0.4699 | 0.8228 | 0.1479 | 0.1116 | 0.0940 |
| **Mean** ± **SEM** | | 0.4432 ± 0.0191 | | | 0.3901 ± 0.1033 | | | 0.5818 ± 0.1206 | | | 0.1179 ± 0.0159 | | |
| **Fold change (Ts1Cje/Wild type)** | | 1.136 | | | | | | 4.9347 | | | | | |
| **p-Value** | | 0.6341 | | | | | | 0.0189* | | | | | |

**Table S4-1: Pixelation analysis of Stat1 bands detected on Western blots in Figure S4.**

**Table S4-2: Pixelation analysis of Ifnar1 and Ifnar2 bands detected on Western blots in Figure S4.**

| **Label** | | **Protein** | **Cerebral cortex** | | | | | | **Cerebellum** | | | | | |
| --- | --- | --- | --- | --- | --- | --- | --- | --- | --- | --- | --- | --- | --- | --- |
|  |  |  | **Ts1Cje** | | | **Wild type** | | | **Ts1Cje** | | | **Wild type** | | |
|  |  |  | **Band 1** | **Band 2** | **Band 3** | **Band 4** | **Band 5** | **Band 6** | **Band 1** | **Band 2** | **Band 3** | **Band 4** | **Band 5** | **Band 6** |
| **S3-1** | | **Ifnar1** | 8089 | 5396 | 12807 | 8509 | 3221 | 4258 | 12874 | 10983 | 18342 | 14576 | 10133 | 7975 |
| **S3-2** | | **Actin** | 6268 | 10827 | 13979 | 17914 | 12758 | 10825 | 5105 | 11412 | 7145 | 11030 | 11987 | 186914 |
| **Ratio A**  **= (S3-1/S3-2)** | | | 1.290 | 0.4984 | 0.9162 | 0.4750 | 0.2525 | 0.3934 | 2.522 | 0.9624 | 2.567 | 1.322 | 0.8454 | 0.0427 |
|  | | | | | | | | | | | | | | |
| **S4-1** | | **Ifnar1** | 1575 | 4819 | 10769 | 6037 | 3554 | 4991 | 13468 | 15146 | 18768 | 9430 | 8559 | 12009 |
| **S4-2** | | **Actin** | 4843 | 2296 | 3308 | 3626 | 2169 | 5070 | 9375 | 4109 | 4506 | 11471 | 7869 | 7613 |
| **Ratio B**  **= (S4-1/S4-2)** | | | 0.3251 | 2.099 | 3.225 | 1.665 | 1.639 | 0.984 | 1.437 | 3.686 | 4.165 | 0.8221 | 1.088 | 1.577 |
|  | | | | | | | | | | | | | | |
| **Average of A & B** | | | 0.8078 | 1.299 | 2.086 | 1.070 | 0.9456 | 0.6889 | 1.979 | 2.324 | 3.366 | 1.072 | 0.9665 | 0.8100 |
| **Mean** ± **SEM** | | | 1.397 ± 0.3722 | | | 0.9015 ± 0.1122 | | | 2.557 ± 0.4168 | | | 0.9494 ± 0.0761 | | |
| **Fold change (Ts1Cje/Wild type)** | | | 1.550 | | | | | | 2.693 | | | | | |
| **p-Value** | | | 0.2711 | | | | | | 0.0192* | | | | | |
| **Label** | **Protein** | | **Cerebral cortex** | | | | | | **Cerebellum** | | | | | |
|  |  |  | **Ts1Cje** | | | **Wild type** | | | **Ts1Cje** | | | **Wild type** | | |
|  |  |  | **Band 1** | **Band 2** | **Band 3** | **Band 4** | **Band 5** | **Band 6** | **Band 1** | **Band 2** | **Band 3** | **Band 4** | **Band 5** | **Band 6** |
| **S5-1** | **Ifnar2** | | 4106 | 3122 | 4656 | 2406 | 1233 | 4375 | 1281 | 2387 | 1917 | 917 | 1765 | 1405 |
| **S5-2** | **Actin** | | 16891 | 13662 | 9990 | 10131 | 9715 | 12442 | 12492 | 13956 | 10687 | 12071 | 16462 | 9208 |
| **Ratio A**  **= (S5-1/S5-2)** | | | 0.2431 | 0.2285 | 0.4660 | 0.2375 | 0.1269 | 0.3516 | 0.1025 | 0.1711 | 0.1794 | 0.0759 | 0.1072 | 0.1526 |
|  | | | | | | | | | | | | | | |
| **S6-1** | **Ifnar2** | | 1240 | 1728 | 4808 | 1606 | 422 | 692 | 906 | 1518 | 1573 | 767 | 638 | 1570 |
| **S6-2** | **Actin** | | 10013 | 10847 | 4847 | 4994 | 5818 | 4072 | 8610 | 9091 | 6864 | 7459 | 12580 | 6164 |
| **Ratio B**  **= (S6-1/S6-2)** | | | 0.1239 | 0.1594 | 0.9920 | 0.3216 | 0.0725 | 0.1699 | 0.1052 | 0.1669 | 0.2292 | 0.1029 | 0.0507 | 0.2546 |
|  | | | | | | | | | | | | | | |
| **Average of A & B** | | | 0.1835 | 0.1939 | 0.7290 | 0.2796 | 0.0997 | 0.2608 | 0.1039 | 0.1690 | 0.2043 | 0.0894 | 0.0789 | 0.2036 |
| **Mean** ± **SEM** | | | 0.3688 ± 0.1801 | | | 0.2133 ± 0.0571 | | | 0.1591 ± 0.0294 | | | 0.1240 ± 0.0399 | | |
| **Fold change (Ts1Cje/Wild type)** | | | 1.729 | | | | | | 1.283 | | | | | |
| **p-Value** | | | 0.4569 | | | | | | 0.5185 | | | | | |

* indicates p-value ≤ 0.05.
